# Exploring the suitability of RanBP2-type Zinc Fingers for RNA-binding protein design

**DOI:** 10.1038/s41598-019-38655-y

**Published:** 2019-02-21

**Authors:** Simona De Franco, Julie Vandenameele, Alain Brans, Olivier Verlaine, Katerina Bendak, Christian Damblon, André Matagne, David J. Segal, Moreno Galleni, Joel P. Mackay, Marylène Vandevenne

**Affiliations:** 1 InBioS-Centre d’Ingénierie des Protéines (CIP), Université de Liège, Liège, 4000 Belgium; 2 Children’s Cancer Institute Lowy Cancer Research, Kensington, 2033 Australia; 3 Laboratoire de Chimie Biologique Structurale (CBS), Département de Chimie, Université de Liège, Liège, 4000 Belgium; 4 Genome Center and Department of Biochemistry and Molecular Medicine, University of California, Davis, CA 95616 USA; 5 School of Life and Environmental Sciences, University of Sydney, Sydney, N.S.W 2006 Australia

## Abstract

Transcriptomes consist of several classes of RNA that have wide-ranging but often poorly described functions and the deregulation of which leads to numerous diseases. Engineering of functionalized RNA-binding proteins (RBPs) could therefore have many applications. Our previous studies suggested that the RanBP2-type Zinc Finger (ZF) domain is a suitable scaffold to investigate the design of single-stranded RBPs. In the present work, we have analyzed the natural sequence specificity of various members of the RanBP2-type ZF family and characterized the interaction with their target RNA. Surprisingly, our data showed that natural RanBP2-type ZFs with different RNA-binding residues exhibit a similar sequence specificity and therefore no simple recognition code can be established. Despite this finding, different discriminative abilities were observed within the family. In addition, in order to target a long RNA sequence and therefore gain in specificity, we generated a 6-ZF array by combining ZFs from the RanBP2-type family but also from different families, in an effort to achieve a wider target sequence repertoire. We showed that this chimeric protein recognizes its target sequence (20 nucleotides), both *in vitro* and in living cells. Altogether, our results indicate that the use of ZFs in RBP design remains attractive even though engineering of specificity changes is challenging.

## Introduction

Recent advances in genome biology have revolutionized our notion of the transcriptome. Over the last past years, RNA has proven to be a main player in eukaryotic biology in many different guises. Alterations in mRNA processing are implicated in different diseases such as Myotonic dystrophy (DM)^1,2^ and cancer^3–5^. In addition, non translated or, so-called, non-coding RNAs (ncRNAs) have emerged as important player of gene regulation and diseases with wide-ranging effects, including in tumors^6–8^ and neurological disorders^9–11^. Therefore, functionalized RNA Binding Proteins (RBPs) that are able to target a chosen RNA sequence would be valuable tools for RNA manipulation. Various protein scaffolds have been trialed for RBP engineering (reviewed in ref.^12^). The most promising data generated to date were obtained with the Pumilio (PUF) proteins. These proteins include eight Pumilio repeats and bind a 8-nt long single-stranded RNA (ssRNA) in a sequence-specific manner through their concave surface. Structural data has permitted the establishment of a complete recognition code for these proteins. In this code, each of the eight repetitions recognizes one base using two amino acid side chains located at specific positions in the repetitions. Specificity changes of PUF proteins can therefore be easily achieved by changing the identities of these residues. Nowadays, RBPs based on PUF domains have been successfully used to manipulate specific RNA targets in living cells^13–15^. However, despite these promising studies, PUF domains exhibit a high repetitive nature, allowing different RNA-binding modes that can lead to off-target binding^16^. Noticeably, the pentatricopeptide repeat (PPR) protein family is also considered as a suitable scaffold for RBP design. Recently, the endonuclease activity of the PPR protein SOT1 has been engineered to target predicted RNA substrates^17^. However, like PUF domains, their repetitive nature and the fact that RNA recognition is limited to one nucleotide per PPR repeat^18,19^ can also promote non-canonical RNA-binding modes that can potentially affect binding specificity^20^.

Zinc-finger (ZF) proteins could potentially represent an attractive alternative candidate for RBP engineering since they are modular, robust to mutations^21^ and they have been successfully used to design DNA-binding proteins. Engineered DNA-binding ZFs were the first proteins used for genome editing and gene therapy^22^ and have been successfully transposed to human therapy^23^. Although, the molecular basis for ssRNA versus double-stranded DNA (dsDNA) recognition are very different, this DNA-binding ZF technology has set good precedent for the use of ZFs to manipulate nucleic acids in the context of living cells as well as full-organisms.

**Figure 1:**
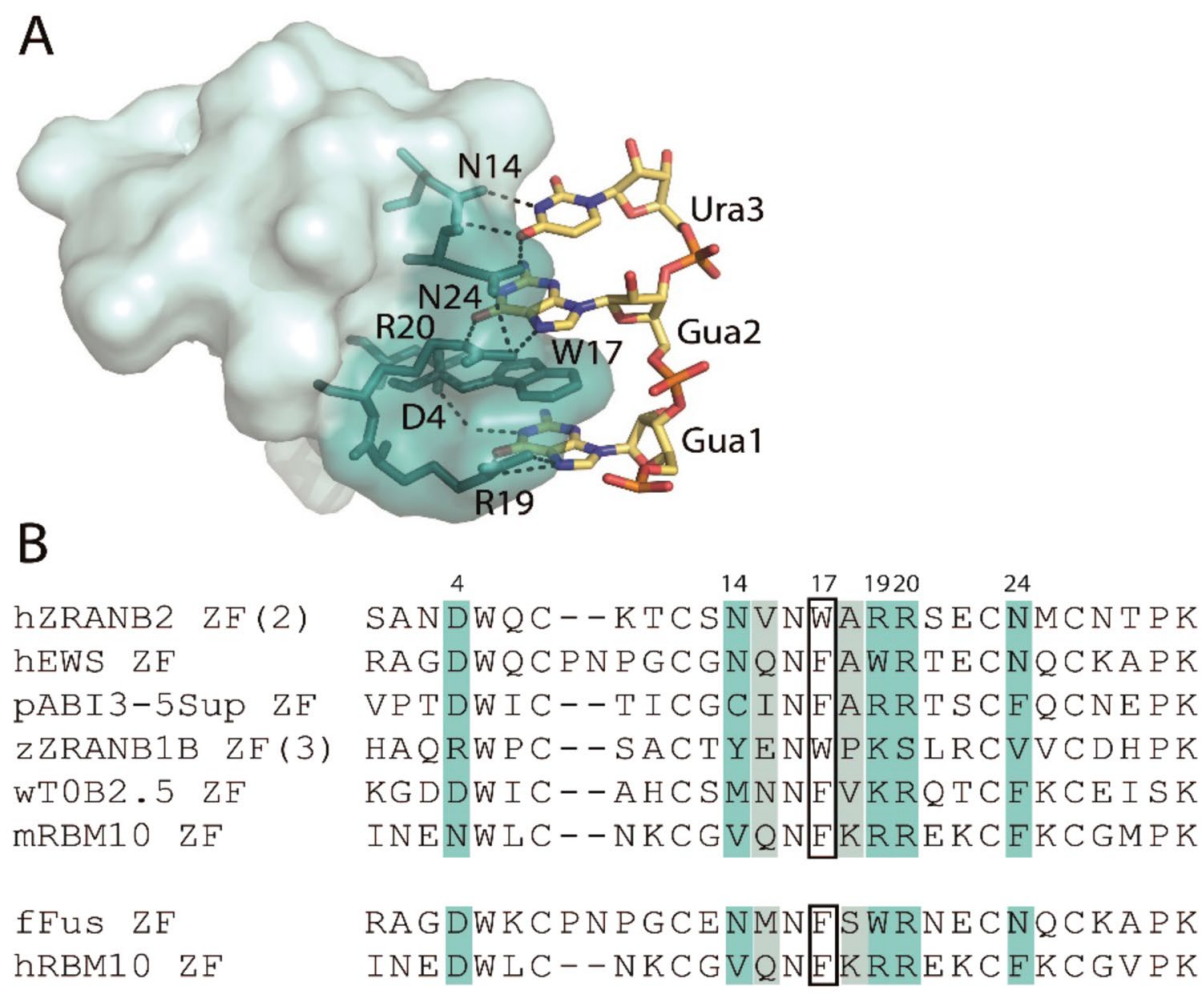
(**A**) X-ray structure of the human ZRANB2-ZF2:ssRNA complex (PDB ID: 3G9Y). Hydrogen bonds are indicated as dashed lines. (**B)** Sequence alignment of ZRANB2 ZF2 and the selected ZFs from different organisms (for details see the Materials and Methods section). Amino acid sequences of FUS and human RBM10 (hRBM10) ZFs are also shown. Marked positions correspond to the RNA-binding residues identified in ZRANB2-ZF2. The tryptophan residue involved in base stacking is enclosed in a box; dark and light blue residues are involved in side chain-mediated and backbone mediated hydrogen bonds with RNA, respectively.

In our case, since we wanted to target ssRNA, we have chosen a particular class of ZFs that naturally recognizes ssRNA with high affinity and specificity. This family, named the RanBP2-type ZF family, was shown to specifically bind to ssRNA^24,25^ and was initially discovered in the human splicing factor ZRANB2. This protein contains two RanBP2-type ZFs (ZF1 and ZF2) and we previously reported that each of these ZFs bind to ssRNA with a μM binding affinity and each of them was shown to target the trinucleotide sequence GGU^24^. The structure of the ZRANB2-ZF2:RNA complex^24,26^ (Fig. 1A) has revealed that sequence-specific recognition is mostly achieved via side-chain mediated contacts with the edges of the RNA bases. We have also shown that modular assembly of these RanBP2-type ZFs into a functional (ZF)_x3_ tandem repeats can be generated^27^. Finally, using a phage-display based combinatorial approach, we showed that specificity changes can be engineered on this ZF family^28^. However, specificity change was successfully achieved only for one trinucleotide sequence (GCC), and in addition the observed binding affinity of the engineered ZF variant was much lower than that of the WT ZF for its WT GGU target^28^.

In the present work, we have adopted an alternative strategy in order to obtain a larger repertoire of recognized RNA sequences. Instead of engineering specificity changes, we evaluated the natural substrate specificity of various members of the RanBP2-type ZF family that, based on the ZRANB2-ZF2:RNA structure, exhibit different RNA-binding residues and, supposedly, bind to different sequences. In addition, the second main goal of this study was to show that we could engineer a functional tandem array of ZF domains in order to target a longer and therefore more specific RNA in the context of a living cell. This is why we generated a (ZF)_x6_ protein that combines RanBP2-type ZFs but also ZFs that belong to other families. We validated this chimeric protein using *in vitro* binding assays as well as a new cell-based assay that we developed in living bacteria.

## Results

### Sequence-specificity analysis of the RanBP2-type ZF family members

**Figure 2:**
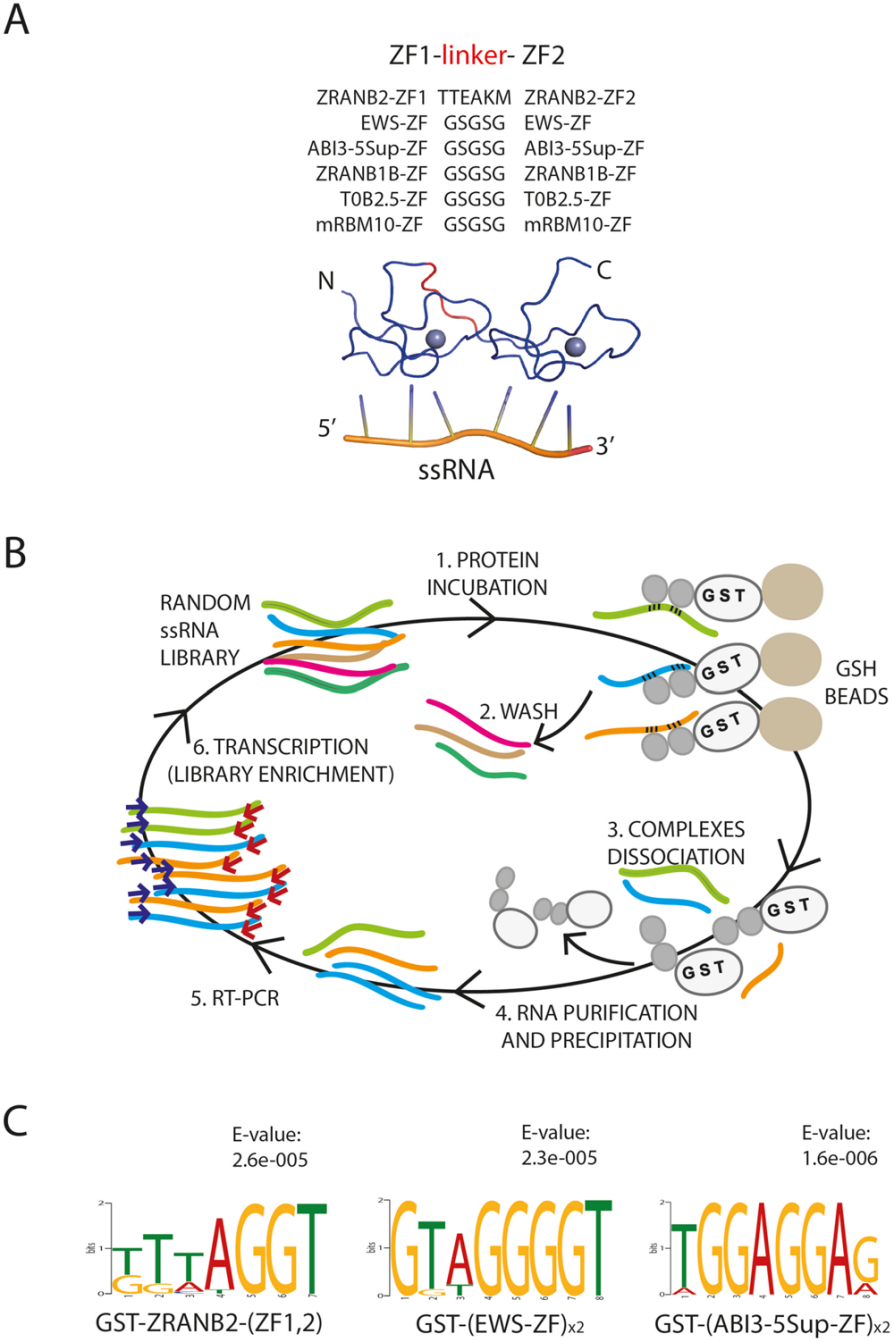
(**A**) Modular constructs employed in SELEX experiments for the selected ZFs and the positive control ZRANB2-(ZF1, 2). (**B**) Schematic representation of the SELEX experiment. (**C**) MEME analysis of SELEX-selected RNA sequences after 4 rounds of selection on ZRANB2-(ZF1, 2), EWS-ZF)_x2_ and (ABI3-5 Sup-ZF)_x2_. The statistical significance (E-value) is shown on the top of each LOGO diagram.

In order to obtain ZF domains that bind to different RNA sequences, we selected five RanBP2-type ZFs for which the likely RNA-binding residues differed from ZRANB2 ZF2; (i) the *Homo sapiens* EWS ZF, (ii) the *Arabidopsis thaliana* ABI3-5Sup ZF, (iii) the *Danio rerio* ZRANB1B ZF, (iv) the *Caenorhabditis elegans* T0B2.5 ZF and (v) the *Mus musculus* RBM10 ZF (Fig. 1B). The selected ZFs were expressed and purified as 2-ZF constructs that consisted of a tandem duplication of the ZF connected by a 5-amino acid linker (GSGSG) and fused to glutathione-S-transferase (GST). In contrast, the ZRANB2-ZFs (ZF1, ZF2) were connected by a shortened version of their natural linker (TTEAKM) (Fig. 2A and Supplementary Fig. S1A), described in our previous work^27^. All proteins were highly expressed, with purification yields reaching ~50–60 mg of GST-tagged (ZF)_x2_ per liter of culture. For each protein, we examined their RNA-binding specificity using Systematic Evolution of Ligands by EXponential enrichment (SELEX)^29^ experiments, in which high-affinity RNA sequences were selected from a random 25 nt-long ssRNA library (Supplementary Table S1) by the GST-fusion proteins pre-immobilized on glutathione-Sepharose beads (Fig. 2B). Noticeably, we preformed the SELEX experiments using 2-ZF constructs just to make sure that the overall binding affinity was sufficient to enrich preferred RNA motifs.

We used the ZRANB2-(ZF1,2) as a positive control since its specificity is known, whereas GST alone provided a negative control. After 4 selection rounds we sequenced 16 cherry picked clones and analyzed them using the motif-based sequence analysis tool MEME (Multiple Em for Motif Elicitation)^30^ (Fig. 2C). MEME analysis on GST-ZRANB2-(ZF1,2) enriched sequences confirmed the expected specificity for GGU motifs. In particular, all the selected sequences exhibited from one to many GGU motifs, leading to a statistical significant consensus (E-value: 2.6 × 10^−5^). For GST-(EWS-ZF)_x2_, the identified consensus (E-value: 2.3 × 10^−5^) indicated specificity of the EWS-ZF for either GGU or GGG motifs. However, we observed a higher occurrence of the GGU over the GGG trinucleotide: 69% of the selected sequences exhibited at least two GGU motifs against the 6% presenting two GGG motifs. For GST-(ABI3-5Sup-ZF)_x2_, 81% of the enriched sequences contained either GGA or GGAGGA motifs. The enriched consensus (E-value: 1.6 × 10^−6^) revealed the specificity of the full (ABI3-5Sup-ZF)_x2_ array for (GGA)_x2_ sequences. No clear enrichment was observed after 4 selection rounds for GST-(ZRANB1-B)_x2_, GST-(T0B2.5)_x2_, GST-(mRBM10)_x2_ or for the GST control (E-values larger than 0.05). Altogether, these data suggest that the characterized ZFs fall into two categories: those that, supposedly do not bind to ssRNA and those that do. In the latter case, they strongly favour GGU or closely related motifs.

### *In vitro* binding assays for the ZF variants against SELEX-selected RNA sequences

To corroborate our SELEX data, we measured the binding affinities of the different ZFs for their selected RNA sequences (Supplementary Table S2) using Isothermal Titration Calorimetry (ITC) and Bio-Layer Interferometry (BLI) (Table 1, Supplementary Figs S3 and S4). In addition to the ZF proteins used in the SELEX experiments, we also conducted the ITC and BLI assays on the FUS ZF (Fig. 1B). This ZF is a murin protein domain whose human homolog was reported to bind to GGU.

**Table 1:** ITC and BLI measurements on the (ZF)_x2_ constructs for their preferred RNA motifs.

(ZFs)_x2_	ssRNA	ITC				BLI		
		n	K_D_ (µM)	∆H° (kcal mol^−1^)	−T∆S° (kcal mol^−1^)	K_D_ (µM)	k_on_ (10^5^ M^−1^ s^−1^)	k_off_ (10^−1^ s^−1^)
ZRANB2	(GGU)_x2_	0.6	0.3 ± 0.08	41.5 ± 2.6	−32.7 ± 2.5	0.3 ± 0.03	3.4 ± 0.1	0.9 ± 0.04
EWS	(GGU)_x2_	0.8	0.2 ± 0.03	25.8 ± 1.5	−16.7 ± 1.4	0.8 ± 0.06	4.5 ± 1.0	3.7 ± 0.6
ABI3-5Sup	(GGA)_x2_	0.6	1.0 ± 0.1	19.5 ± 0.1	−11.3 ± 0.2	2.6 ± 1.0	1.8 ± 0.3	4.3 ± 1.6
ABI3-5Sup	(GGU)_x2_	0.9	0.7 ± 0.1	24.3 ± 1.5	−15.8 ± 1.7	1.0 ± 0.01	2.9 ± 0.01	2.9 ± 0.1
FUS	(GGU)_x2_	0.7	0.2 ± 0.02	35.8 ± 7.2	−26.7 ± 7.0	0.8 ± 0.07	3.5 ± 0.3	2.9 ± 0.5

Affinities are presented as equilibrium dissociation constants (K_D_). Reported values correspond to the means and associated standard errors calculated from at least two independent experiments. ITC and BLI measurements were carried out at 25 °C and 30 °C, respectively.

All ZFs were expressed and purified as GST-(ZF)_x2_ proteins. After cleavage of the GST tag, we isolated the (ZF)_x2_ arrays by size exclusion chromatography, with an average yield of 8 mg of purified and isolated (ZF)_x2_ per liter of culture (Supplementary Fig. S2). Binding experiments were carried out using the purified (ZF)_x2_ proteins, as described in the experimental section.

ITC data revealed that both (EWS-ZF)_x2_ and (FUS-ZF)_x2_ bound to (GGU)_x2_ with equilibrium dissociation constants (K_D_) similar to that observed for ZRANB2-(ZF1,2) (Table 1, Supplementary Fig. S3). Surprisingly, (ABI3-5-Sup-ZF)_x2_ showed a similar affinity for a (GGU)_x2_ motif compared to the SELEX-selected (GGA)_x2_ motif. As expected, no binding to RNA (using GGU or polyA control sequences) could be detected for either (ZRANB1-B-ZF)_x2_ and (T0B2.5-ZF)_x2_ constructs, whereas a lower affinity binding (K_D_ = ~10 µM) with (GGU)_x2_ was observed for (mRBM10-ZF)_x2_ (Supplementary Fig. S3).

It is interesting to note that all the binding reactions measured by ITC were always characterized by large favorable enthalpy changes and unfavorable entropy changes, as observed for other ssRNA-binding domains^31^. As expected, the values of binding reaction stoichiometry are close to 1:1 or a bit lower, likely due to difficulties in accurate estimation of RNA or protein concentration, as described in our previous study^32^. Overall the BLI and ITC measurements are in good agreement (Table 1, Supplementary Figs S3 and S4), with all dissociation constants in the low μM range. The binding kinetics (k_off_ and k_on_) were also closely similar for all the studied ZFs.

### Importance of position 4 in the RNA-binding platform of RanBP2-type ZFs

**Figure 3:**
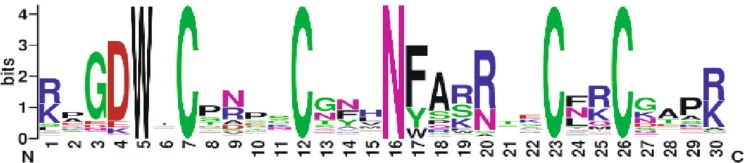
WebLogo diagram representing the conserved amino acidic positions among putative RNA-binding RanBP2-type ZFs. We selected the RanBP2type ZFs (accession code pfam00641) exhibiting an aromatic residue at position 17 that could potentially be stacked between the RNA bases, as observed for W17 in ZRANB2 ZF2: RNA structure. Among all the conserved residues, D4 was present in almost 80% of the sequences.

Interestingly, the mouse RBM10 ZF variant (mRBM10-ZF) that we studied herein exhibited no enrichment in SELEX and presented a lower binding affinity for GGU motifs compared to the other studied ZFs that were shown to bind RNA. This observation is intriguing given that this protein only differs by 2 amino acids (at position 4 and 26) from the human RBM10 ZF (Fig. 1), which was previously shown to bind to a GGG motif ^25^ with low μM binding affinity. In order to analyze the importance of these two residues in the RNA-binding affinity, we generated single mutations (N4D, M26V) in the mRBM10-ZF variant. The corresponding mutated proteins and the RBM10 ZFs were expressed and purified as isolated (ZF)_x2_ arrays (Supplementary Fig. S1B). Then, we measured the affinities of each protein for the target sequence GGG (Table 2 and Supplementary Fig. S5). As expected, (mRBM10-ZF)_x2_ exhibited a lower affinity (five-fold) compared to its human homolog (hRBM10-ZF)_x2_. Interestingly, the single N4D mutation was sufficient to restore the RNA binding affinity measured for (hRBM10-ZF)_x2_, whereas M26V had no observable effect on the RNA-binding affinity, suggesting that D4 was solely responsible for the difference in binding affinity between the RBM10 ZF homologs. Furthermore, this residue seems to be highly conserved among the putative RNA-binding RanBP2-type ZFs (Fig. 3) since 80% of the sequences exhibit a Asp (D) residue in position 4, the other 20% of the sequences harbor either a Lys (K) or a Glu (E) residue. This later observation has been further investigated and discussed in the Supplementary Data (Fig. S6).

**Table 2:** BLI measurements of the RBM10 variants/mutants binding to a (GGG)_x2_ RNA. Reported means and associated standard errors were calculated from two independent experiments.

(ZFs)_x2_	ssRNA	K_D_ (µM)	k_on_ (10^5^ M^−1^s ^−1^)	k_off_ (10^−1^ s^−1^)
hRBM10	(GGG)_x2_	0.3 ± 0.03	4.6 ± 0.8	1.4 ± 0.1
mRBM10	(GGG)_x2_	2.2 ± 0.05	2.1 ± 0.2	4.7 ± 0.4
N4D	(GGG)_x2_	0.4 ± 0.03	4.4 ± 0.8	1.8 ± 0.2
M26V	(GGG)_x2_	2.8 ± 0.3	1.8 ± 0.1	4.9 ± 0.8

### Analysis of the discrimination abilities of the ZF variants

BLI experiments were also conducted to analyze the discrimination ability of the different studied (ZF)_x2_ proteins towards RNA sequences in which single mutations were introduced (Table 3) in the first, second and third positions of the recognized trinucleotide motif. We performed the G → A mutation for the first and the second positions whereas for the third position the U_3_ → A/G/C mutations were performed in order to assess whether ZFs other than ABI3-5-Sup also exhibited tolerance to modifications of the third base. For all the studied (ZF)_x2_, mutation of any guanine in the first and second positions of the GGX motif completely abolished the RNA-binding activity (Table 3). In contrast, the ZFs exhibited different behaviors when the third base was mutated, depending also on the replacing nucleotide (Table 3, Supplementary Fig. S6). For ZRANB2-(ZF1,2), (EWS-ZF)_x2_, and (FUS-ZF)_x2_, RNA binding was only observed in the case of the U_3_ → G substitution. However, the binding affinity for a GGG motif was 2.3-fold reduced for ZRANB2-(ZF1,2) and 1.3-fold reduced for both (EWS-ZF)_x2_ and (FUS-ZF)_x2_ compared to the GGU sequence. Interestingly, (ABI3-5Sup-ZF)_x2_ and (hRBM10-ZF)_x2_ showed higher tolerance to modifications of the third base. Despite a 3-fold preference for U in the third position, (ABI3-5Sup-ZF)_x2_ bound to GGG as well as to GGA motifs. In contrast, protein binding to GGC RNA was too low to allow accurate fitting, therefore we could not report affinity for this sequence. Finally, although (hRBM10-ZF)_x2_ bound to all tested GGN sequences, it showed a preference for GGG over GGU (1.6-fold), GGA (4.6-fold) and GGC (8.6-fold) motifs.

**Table 3:** BLI results obtained for each (ZF)_x2_ with all tested RNAs.

	ZRANB2-(ZF1,2)			(EWS-ZF)_x2_			(FUS-ZF)_x2_			(ABI3-5Sup-ZF)_x2_			(hRBM10-ZF)_x2_		
	K_D_ (µM)	k_on_ (10^5^ M^−1^s^−1^)	k_off_ (10^−1^ s^−1^)	K_D_ (µM)	k_on_ (10^5^ M^−1^s^−1^)	k_off_ (10^−1^ s^−1^)	K_D_ (µM)	k_on_ (10^5^ M^−1^ s^−1^)	k_off_ (10^−1^ s^−1^)	K_D_ (µM)	k_on_ (10^5^ M^−1^s^−1^)	k_off_ (10^−1^ s^−1^)	K_D_ (µM)	k_on_ (10^5^ M^−1^s^−1^)	k_off_ (10^−1^ s^−1^)
(GGU)_x2_	0.3 ± 0.03	3.4 ± 0.1	0.9 ± 0.04	0.8 ± 0.06	4.5 ± 1.0	3.7 ± 0.6	0.8 ± 0.07	3.5 ± 0.3	2.9 ± 0.5	1.0 ± 0.01	2.9 ± 0.01	2.9 ± 0.1	0.5 ± 0.01	2.5 ± 0.1	1.3 ± 0.05
(AGU)_x2_	NB			NB			NB			NB			NB		
(GAU)_x2_	NB			NB			NB			NB			NB		
(GGA)_x2_	NB			NB			NB			2.6 ± 1.0	1.8 ± 0.3	4.3 ± 1.6	1.4 ± 0.01	1.4 ± 0.02	1.9 ± 0.02
(GGC)_x2_	NB			NB			NB			*			2.6 ± 0.03	1.9 ± 0.02	4.9 ± 0.004
(GGG)_x2_	0.7 ± 0.01	4.9 ± 0.06	3.2 ± 0.01	1.1 ± 0.01	9.3 ± 0.1	10.3 ± 0.1	1.1 ± 0.3	2.9 ± 0.04	3.3 ± 0.02	3.0 ± 0.5	1.7 ± 0.02	5.2 ± 0.01	0.3 ± 0.03	4.6 ± 0.8	1.4 ± 0.1
(AAA)_x2_	NB			NB			NB			NB			NB		

Reported means and associated standard errors were calculated from at least two independent experiments. “NB” stands for “No Binding”; “*” stands for weak and not fitted binding between (ABI3-5Sup-ZF)_x2_ and (GGC)_x2_ RNA.

### Design and characterization of a (ZF)_x6_ array

**Figure 4:**
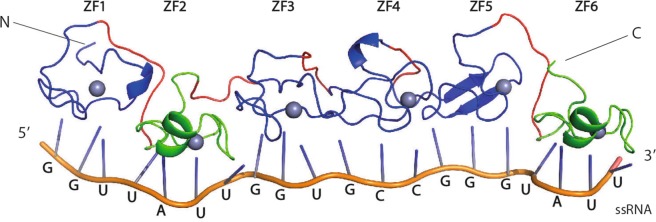
Schematic representation of the (ZF)_x6_ array. From the left: the EWS RanBP2-type ZF, the second CCCH-type ZF of Ts11d, the second RanBP2-type ZF of ZRANB2, a phage display ZRANB2 ZF2, the RBM5 RanBP2-type ZF, the first CCCH-type ZF of Ts11d. The RanBP2-type ZFs and the CCCH-type ZF are blue and green, respectively. Linker regions are shown in red.

In order to target a longer RNA sequence and therefore increase the specificity of our ZF arrays in cell-based applications, we generated a tandem array of 6 ZFs. In addition, since the sequence repertoire recognized by RanBP2-type ZFs is restricted to GGN motifs, we decided to combine RanBP2-type ZFs with ZFs from a different family (CCCH-type ZFs) that were shown to bind ssRNA with different sequence specificity. The designed protein sequence included from N- to C-terminal: the EWS RanBP2-type ZF, the Ts11d CCCH-type ZF2^33^, RanBP2-type ZRANB2 ZF2, a phage display mutant of ZRANB2 ZF2^28^, the RBM5 RanBP2-type ZF^27^ and the Ts11d CCCH-type ZF1^33^ (Fig. 4 and Supplementary Fig. S1C).

**Figure 5:**
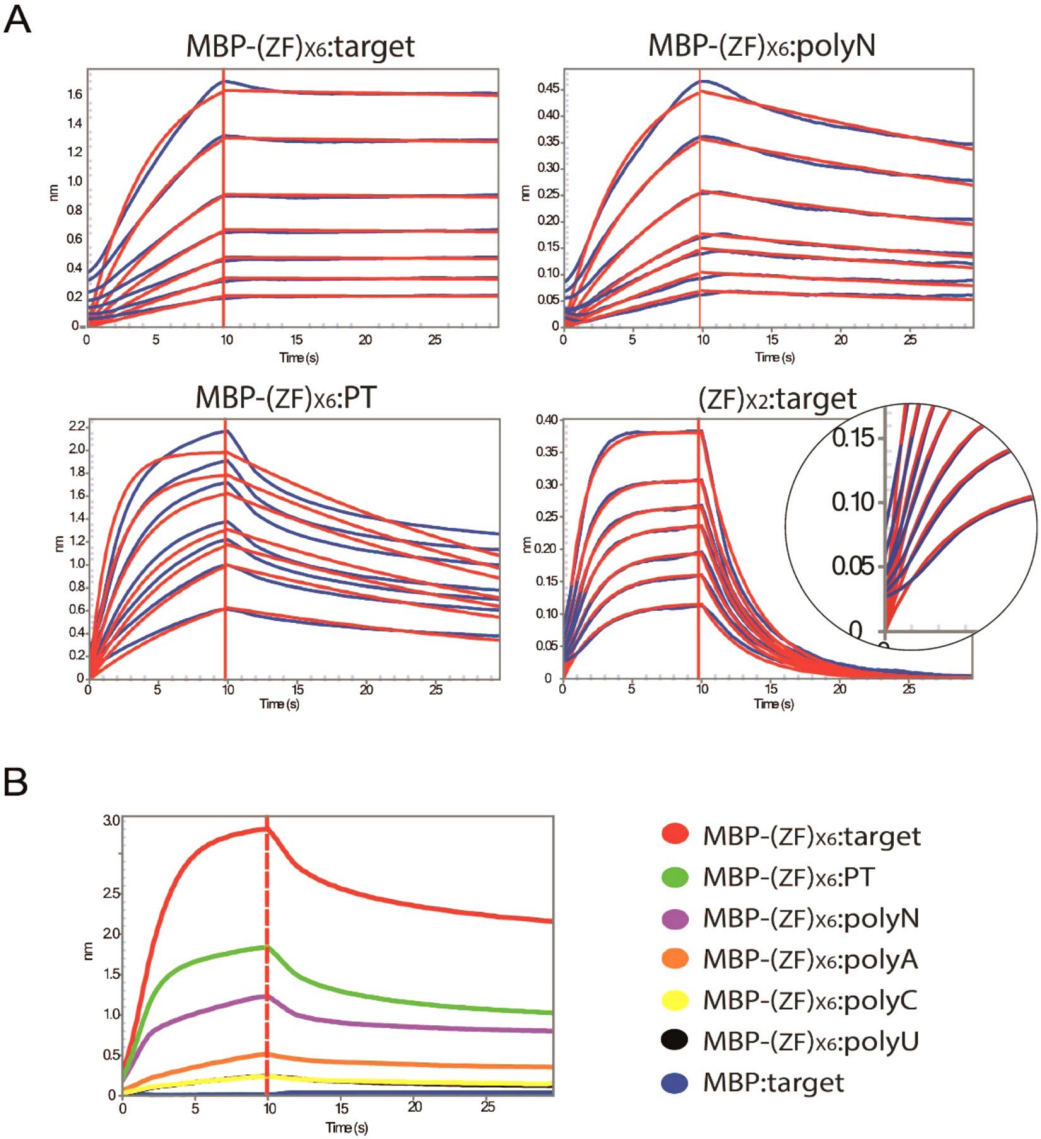
BLI experiments on the MBP-(ZF)_x6_ protein. (**A**) Sensograms (blue) showing the binding of the chimeric protein to its target RNA, to a library of random sequence (polyN), and to a permuted target (PT). The corresponding fit (simple 1:1 model (red)) presents reduced quality that is mostly attributed to the sigmoid-like early association phase. This behavior was also observed but to a smaller extent in the kinetic profiles of the different (ZF)_x2_:RNA interactions (e.g., bottom-right panel). This phenomenon could results from a rapid adsorption of the proteins to the sensor surface at the very beginning of association phase, which causes a non-zero initial signal. This behavior may be amplified in the case of the chimera because of the larger number of ZFs assembled and their different affinities for target motif. (**B**) Comparison of BLI experimental and control data collected at a protein concentration of 5 µM.

In contrast to the GGU-specific EWS and ZRANB2 ZFs, the ZRANB2 ZF2 mutant and RBM5 ZF recognized GCC and GGG motifs, respectively^27,28^. Both Ts11d ZFs bound to a UAUU motif^34^. The (ZF)_x6_ array was expressed in fusion with the Maltose Binding Protein (MBP) and purified by affinity chromatography followed by size exclusion chromatography. On average, we recovered **~**20 mg of pure MBP-(ZF)_x6_ per liter of culture (Supplementary Fig. S9). We worked with the full MBP-fusion protein to overcome protein stability and solubility issues. Using BLI, we showed that this MBP-(ZF)_x6_ protein bound its 20-nt long target RNA (GGU UAUU GGU GCC GGG UAUU) with an affinity of 24 ± 0.6 nM (Table 4 and Fig. 5A) suggesting that the binding affinity of this (ZF)_x6_ protein is ≈26-fold tighter compared to the original (ZF)_x2_ constructs. Since we previously reported that the addition of a third RanBP2-type ZFs to a 2-ZF construct led to a 4-fold increase of binding affinity for the target RNA^27^, this observation suggests that the ZFs included in this (ZF)_x6_ chimeric protein are at least partially functional.

**Table 4:** BLI-derived kinetic parameters of MBP-(ZF)_x6_ binding to RNA.

ssRNA	K_D_ (nM)	k_on_ (10^4^ M^−1^s^−1^)	k_off_ (10^−3^ s^−1^)
Target	24 ± 0.6	5 ± 0.1	1 ± 0.1
polyN	275 ± 55	7 ± 2	17 ± 3
PT	223 ± 17*	14 ± 0.6*	30 ± 1*

Reported means and associated standard errors were calculated from at least two independent experiments. Kinetic parameters for PT RNA (asterisk) were only indicative because the one-site model did not provide accurate data fit (Fig. 5A).

To assess the binding specificity of this generated (ZF)_x6_ protein, we measured the binding of the protein binding to a library of random 20 nt-long RNA sequences (polyN) as well as to an RNA in which the 3/4-nt subsites were permuted to give the sequence: UAUU GGU GGG UAUU GCC GGU (PT RNA). Our data indicated that control RNAs bound with only a 10-fold reduced binding affinity compared to the designed target (Table 4 and Fig. 5A). In particular, the (ZF)_x6_ protein associated to its target and also to polyN RNAs with rate constant (k_on_) values that are identical within the error limit, whereas the dissociation was 17-fold faster for random RNAs compared to the target. Noticeably, the binding kinetic profiles measured for the permuted target RNA (PT RNA) were more complex with increased non-specific contributions (plateau drift, Fig. 5).

Finally, BLI experiments conducted on polyU, polyA and polyC RNA controls resulted in very low signal intensities that we attributed to nonspecific binding (Fig. 5B). Other binding experiments were performed against control molecules (ssDNA and dsDNA) and are presented and discussed in the Supplementary Data (Fig. S7).

### Assessment of the binding specificity of the (ZF)_x6_ protein in a living cell

**Figure 6:**
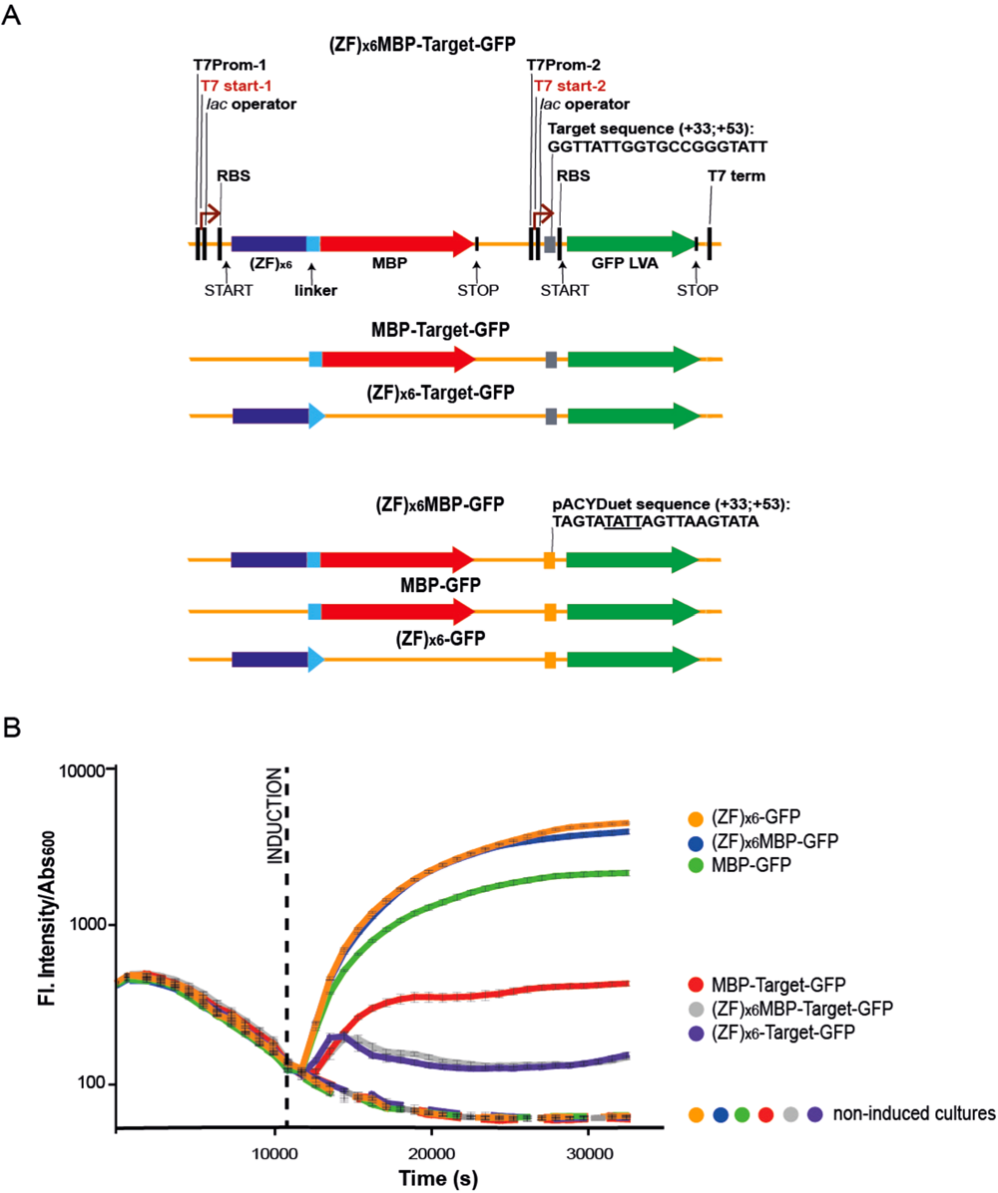
(**A**) Representation of the vectors employed in the RNA-binding bacteria-based assay. Sequence features are detailed for the initial vector (ZF)_x6_MBP-Target-GFP vector. The sequence coding for the target replaced the plasmid region upstream the second RBS (+33; +53); the excision of (ZF)_x6_ and MBP CDSs yielded to the vectors MBP-target-GFP and (ZF)_x6_-target-GFP, respectively. The pACYDuet sequence at positions from +33 to +53 remained unchanged in the control plasmid (ZF)_x6_MBP-GFP and its derivatives MBP-GFP and (ZF)_x6_-GFP. Importantly, the unmodified region +33 to +53 contains a TATT motif that codifies one of target subsites (underlined). (**B)** Fluorescence emission over time for one of the two independent experiments performed in sextuplicate. Collected data were normalized by dividing for the absorbance at 600 nm monitored over time. Reported values correspond to the means and associated standard errors of each repeat. Dashed lines indicate non-induced control cultures. Vectors carrying the target sequence upstream the RBS exhibit reduced fluorescence intensity. This behavior was quite expected, considering that target assembling modified the optimized RBS region of the commercial plasmid.

Based on the *in vitro* binding assays performed with the (ZF)_x6_ chimeric protein, we asked whether the 17-fold slower dissociation rate observed for the target RNA compared to random RNA sequences would be sufficient to see the specific binding of the (ZF)_x6_ protein to its target RNA in a living cell. For this purpose, we designed a new bacteria-based assay. In these experiments, we used genetic constructs derived from the pACYDuet vector for dual expression of two gene cassettes (Fig. 6A). We generated three different plasmid constructs with the first gene cassette encoding either: i) the (ZF)_x6_ array fused to a C-terminal MBP-tag; ii) the (ZF)_x6_ array alone or iii) the MBP alone. In all these constructs, the second gene cassette coded for a variant of the green fluorescent protein (GFP LVA) that exhibited a reduced half-life when expressed in *Escherichia coli* compared to normal GFP^35^. Importantly, upstream of the ribosome-binding site (RBS) in the second cassette, we replaced the vector sequence corresponding to positions +33 to +53 with the sequence coding for the 20 nt-long target RNA. These three vectors were named: (ZF)_x6_-MBP-target-GFP, (ZF)_x6_-target-GFP and MBP-target-GFP vector (Fig. 6A). In addition, we generated a control set of plasmids that included the same gene cassettes with the exception that the sequence upstream the RBS was not modified. These plasmids were termed: (ZF)_x6_-MBP-GFP, (ZF)_x6_-GFP, MBP-GFP. The idea behind this new bacteria-based assay is that specific interaction of the (ZF)_x6_ chimeric protein with its target sequence would interfere with proper assembly of the ribosome machinery and consequently reduce GFP translation levels. In other words, this experiment can be seen as a cell-based assay to study the binding specificity of the chimeric protein where the (ZF)x6 has to locate its target RNA sequence among an ocean of RNAs that are present in the bacteria and compete with specific binding.

It is also important to mention that we chose bacteria and not eukaryotic cell-based assays for several reasons: i) bacteria do not have any cell compartment (no nucleus), therefore all the RNAs present in the cell are available as competing molecules, and ii) bacteria antibiotic resistance has always been the main interest of our lab and our long-term goal is to be able to target regulatory RNAs involved in antibiotic resistance^36^ using our ZF technology.

For each construct, we monitored the expression of GFP LVA in living *E. coli* krx cells over time, after IPTG and L-rhamnose induction (Fig. 6B). For plasmids carrying the target RNA sequence, we observed that the expression of the RBP (either with the MBP-tag or alone) was accompanied by a decrease in fluorescence intensity. In contrast, when the MBP was expressed alone, fluorescence production gradually increased over time. Similarly, the GFP LVA protein was expressed at high levels in all bacteria transformed with the plasmids from control set, in which the region adjacent to the RBS was not modified. Altogether these data suggest that our (ZF)_x6_ array is specific enough to discriminate its target RNA sequence in the context of the crowded environment of a living bacteria.

It is also important to note that all the bacteria transformed with the plasmid encoding the (ZF)_x6_ protein (alone or in fusion with MBP) exhibited normal growth behavior and phenotype which suggested that non-specific binding, if present, was negligible and compatible with the bacteria normal metabolism and growth.

## Discussion

In this study, we have attempted to engineer functional RanBP2-type ZFs tandem arrays in an effort to generate tools for RNA study and manipulation. First, we have focused our work on extending the sequence specificity repertoire of the RanBP2-type ZF family by analyzing the substrate specificities of various ZF members of this family that presented different amino acid composition on the predicted RNA-binding surface. Our SELEX experiments and binding assays have revealed that these ZF domains exhibit an unexpected highly conserved sequence specificity for GGU/GGN motifs. Since their associated full-length proteins are mostly predicted to be splicing factors^37–41^, and because the GGU motif resembles the 5′ splice site consensus motif, the redundant specificity of ZFs may reflect the high degree of conservation at splice site junctions and regulatory sequences. ZRANB2 has been proposed to recognize 5′ splice sites characterized by the consensus AG│GUR (R = purine) in mammals^24^. It is therefore plausible that EWS, FUS, ABI3 and RBM10 carry out a similar function. In addition, splicing intronic/exonic enhancers like UGGG repeats^42^, G-triplets^43,44^, GGAGA^45,46^, UAGG and GGGG^47^ sequences are close to the SELEX-selected motifs and therefore could be the targets of these proteins as well.

Furthermore, a critical interaction for RNA recognition in the ZRANB2-ZF2:RNA structure is the hydrogen bond network formed between both guanines and two arginine sidechains (R19, R20; Fig. 1A)^24^. Both residues are conserved in RBM10 ZF whereas EWS and FUS ZFs showed only conservation of R20. Notably, this substitution of R19 for tryptophan in EWS and FUS ZFs did not appear to affect the RNA specificity. We hypothesized that this side chain can potentially stack with the guanine base, perhaps compensating for the loss of the R19-mediated hydrogen bonds. In contrast, in the ZF from the zebrafish protein ZRANB1-B, which did not bind RNA, a lysine and a serine replace R19 and R20, respectively. These amino acid substitutions reduce the number of possible hydrogen bonds and preclude any compensatory stacking interaction. It is important to note that this ZF shares high sequence conservation with its human counterpart that is reported not to bind RNA but ubiquitin^48–50^. In contrast, we did not identify any specific feature that could explain the inability of the ZF from the worm protein T0B2.5 to bind to RNA. The R19K exchange found in T0B2.5 ZF is also found in its human homolog RBM5 that is able to bind to GGG RNA^27^. Furthermore, T0B2.5 and RBM5 ZFs share all putative RNA-binding residues, with the only exception of position 14, a residue that contacts the third nucleotide in the ZRANB2 ZF2:RNA structure. However, these two homologs exhibit poor conservation of non RNA-binding residues; therefore conformational changes may explain their opposite RNA-binding behaviors.

In this work, we have also analyzed the RNA-binding properties of the human and mouse variants of RBM10-ZF. Our mutagenesis results highlighted the importance of residue D4 in the binding affinity of RanBP2-type ZFs and showed that an aspartate at this position is responsible for a 5-fold contribution to RBM10 ZF RNA-binding affinity. The computational analysis of putative RNA-binding RanBP2 type ZFs sequences indicated that this residue is highly conserved, suggesting a key role in RNA binding. In the ZRANB2 ZF2:RNA structure, D4 forms a water-mediated hydrogen bond via its carboxylate group with an imino proton of the second guanine^24^. We hypothesized that replacement of D4 with the neutral isosteric asparagine could destabilize this hydrogen bond.

Our data have also shown that all the studied ZFs are highly discriminative for the dinucleotide G_1_G_2_. In contrast, modifications of the third nucleotide seemed to be better tolerated. The ZRANB2, EWS and FUS ZFs only bind to GGU or GGG motifs and appeared to be the most discriminating. We hypothesized that the conservation of the carbonyl groups (C=O) on this third base can explain this behavior. Indeed, in the ZRANB2 F2:GGU structure (Fig. 1), both carbonyl groups of U_3_ are specifically recognized by two asparagines (N14, N24) that are conserved in EWS and FUS ZFs. In contrast, ABI3-5Sup-ZF and hRBM10-ZF, who tolerate any base on third position, exhibit substitution of both residues N14 and N24 which reduces the number of possible hydrogen bonds with the third nucleotide. For ABI3-5Sup ZF, the N14 is replaced by C14 that could only form a single hydrogen bond via its thiol group, whereas F24 could potentially stack any base. In hRBM10 ZF, hydrophobic residues replaced both asparagines (N14 → V; N24 → F), which explains the reduced specificity on the third base. Interestingly, both of these ZFs are surrounded by RRM domains in the full-length protein (Supplementary Fig. S11), which could partially reduce promiscuous RNA binding. It is also possible that this less discriminative behavior could allow ZFs to bind to various sequences while searching/scanning RNAs, before specific recognition by the full-length protein.

Altogether, these *in vitro* binding measurements have shed new lights on the molecular bases that drives ssRNA:RanBP2-type ZF interactions.

In this study, we also generated for the first time a (ZF)_x6_ array in order to be able to target a longer RNA sequence, and therefore increase the specificity of our ZF proteins for future applications in cells. Given that RanBP2-type ZFs present a restricted sequence repertoire of RNA targets, we decided to combine RanBP2-type ZFs with ZFs from other families that were shown to bind RNA and target different sequences. This chimeric (ZF)x6 can be seen as a proof of concept experiment that shows that we can combine ZFs from different families in an effort to extend the sequence-specificity repertoire of the generated ZF arrays. Our *in vitro* binding assays indicated that the resulting 20-kDa protein exhibited a ≈ 25 nM affinity for its 20-nt long target RNA. These data are very encouraging since higher affinity (low nM) RNA-binding domains such as PUF and PPR repeats include eight repeats that are 35/36-residues long and each of these repeats specifies and interact with a single nucleotide, which means that a 70-kDa protein would be required to target a 20-nt long sequence.

The analysis of the *in vitro* binding specificity of our chimeric (ZF)_x6_ protein revealed a relatively poor discrimination ability between cognate and non-cognate RNAs (Table 4 and Fig. 5A).

This is why, we went on to determine whether the observed 20-fold affinity difference was sufficient to provide specific binding in a living cell. For this purpose, we designed a new cell-based assay in bacteria (*E.coli*) using a dual expression plasmid encoding our (ZF)_x6_ protein (alone or in fusion with MBP) and the GFP, with the region upstream its RBS replaced by the sequence encoding the target RNA. Our data suggest that in a living cell, our (ZF)_x6_ protein is able to bind its cognate RNA sequence and thereby decrease the yield of expressed GFP, most likely by diminishing the accessibility of the RBS to the translational machinery.

Although additional *in vivo* experiments should be done to characterize the RNA-binding specificity of the (ZF)_x6_ array as well as the contribution of each zinc finger to the modular recognition of protein target, this new bacteria-based assay offers a quick and simple approach to assess any protein-RNA interaction *in vivo*.

It is also interesting to mention that, although, the sequence specificity repertoire of the individual ZF domains that we studied herein is limited, by combining these ZFs in different ways, it is possible to target numerous RNA sequences. Indeed, the different 20 nt-long RNA sequences that we could target by combining all the ssRNA-Binding ZFs known up to date (**GGU**: ZRANB2-ZF1, ZRANB2-ZF2, EWS-ZF, FUS-ZF, ABI3-5-Sup-ZF/**GCC**: ZRANB2-ZF2-HN/**GGG**: RBM5-ZF, RBM10-ZF/**UAUU**: Ts11d-ZF1, Ts11d-ZF2) is estimated to be close to ≈4^6^ = 4096, namely 4096 possible ssRNA targets. This rough estimation makes the assumption that we can combine 4 different target subsites (3 or 4 nt- long) to create these 20 nt-long target sequences.

Altogether, our study comforts the idea that RNA targeting by ZFs-arrays remains quite attractive. Indeed, our ZF-based approach is relatively simple and mainly relies on the functional assembly of ZFs units (from the same or different families) that can be fused to many different effector domains (translational regulator, RS domains…) and therefore constitutes a versatile tool for RNA study and manipulation. In addition, ZF-based proteins can be easily delivered, expressed under the regulation of various tissue-specific promoters or addressed to specific cellular compartments via specific signal peptides. However, the main limitation of this technology lies on the restrained sequence repertoire targeted by RanBP2-type ZFs. The RanBP2-type ZFs only recognize GGU or GGN motifs despites our previous engineering attempt that led to the generation of a single ZF variant displaying specificity (but reduced affinity) for a GCC sequence^28^. More efforts should be done to elucidate the rules that govern RNA recognition by RanBP2-type ZFs and other ZF families in order to improve specificity-engineering approaches. A universal recognition code may not be achieved but at least few engineered ZFs or ZFs from various families could find a direct and easy application *in vivo*.

## Methods

### Preparation of the (ZF)_x2_ proteins

GenBank accession codes and residues from the RanBP2-type ZF variants chosen for analysis are as follows: ZRANB2 (*H. sapiens*), NP_976225, residues 8–38 (ZF1) and 65–95 (ZF2); EWS (*H. sapiens*), NP_053733, residues 523–555; RBM10 (*M. musculus*), NP_001155288, residues 135–166; Fus (*X. laevis*), NP_001080383, residues 435–467; ZRANB1-B (*D. rerio*), NP_001071236, residues 152–182; T08B2.5 (*C. elegans*), NP_871822, residues 86–116; ABI3–5 Suppressor (*A. thaliana*), NP_001190084, residues 379–406. Zinc finger domains were duplicated and covalently linked by a 5 amino acids linker (GSGSG). In contrast, the ZRANB2 fingers (F1, 2) were connected by a shortened version of their natural linker, previously optimized (TTEAKM)^27^. Genes encoding these constructs were commercially purchased (*GeneCust*, Luxembourg) and cloned into the expression vector pGEX6P-1. The integrity of the genetic constructs were confirmed by DNA sequencing. Protein expressions were carried out in *E. coli* (DE3) Rosetta cells at 18 °C (OVN) upon induction with 0.1 mM IPTG and supplementation with 0.1 mM ZnCl_2_. Cell lysis was performed using a cell destroyer equipment (Emulsiflex C3, Avestin, Europe GmbH, Germany) in 50 mM Tris-HCl pH 8, 1 M NaCl, 1 mM dithiothreitol (DTT), 0.1 mM ZnCl_2_ and one Complete EDTA-free protease inhibitor mixture tablet (Roche Applied Science). GST fusion proteins from the soluble fraction were purified on Glutathione-Sepharose^®^ 4B beads (GE Healthcare) and eluted using a 100 mM reduced gluthation solution. The GST tags were cleaved off the (ZF)_x2_ constructs using HRV-3C (OVN, 4 °C) in 50 mM Tris pH 8, 150 mM NaCl, 1 mM DTT and 0.1 mM ZnCl_2_ and all (ZF)_x2_ constructs were further purified by gel filtration (Sephacryl HR100, XK16/70-120 mL). Protein folding was analyzed by both intrinsic fluorescence and one-dimensional ^1^H NMR spectroscopy.

### Preparation of the (ZF)_x6_ chimeric protein

The designed 6-ZFs chimeric protein (Fig. 1C) includes (from the N- to the C-terminus) the following ZFs: EWS, Ts11d F2 (P47974, residues 191–219), ZRANB2 F2, ZRANB2 F2 (HN mutant)^28^, RBM5 (NP_005769, residues 181–211), Ts11d F1 (P47974, residues 153–181). All these domains were connected by a 5 amino acids linker (GSGSG), excepted for those followed by classical fingers. In this latter case, four additional residues (GSGSGSGSG) were introduced in order to reduce potential steric hindrances. The choice of the selected ZFs required consistency in domain polarities since our ZFs bind to ssRNA in a directional manner (in our case: N → Cter to 5 → 3′). The gene encoding this chimeric protein was purchased (*GeneCust*, Luxembourg) and cloned into the pMALC2X vector. Protein expressions were carried out in *E. coli* (DE3) Rosetta cells at 18 °C for 4–5 hours upon induction with 0.1 mM IPTG and supplementation with 0.1 mM ZnCl_2_. Cells were lysed by sonication in 20 mM Tris-HCl pH 7.5, 150 mM NaCl, 1 mM DTT, 0.1 mM ZnCl_2_ and one Complete EDTA-free protease inhibitor mixture tablet (Roche Applied Science). The MBP fused protein from the soluble fraction was purified on Amylose resin (NewEngland Biolabs) and eluted using a 10 mM maltose solution. An additional step of purification by gel filtration (Sephacryl HR100, XK16/70-120 mL) was carried to ensure size-homogeneity in the final sample.

### Image acquisition and processing

SDS-PAGE data were acquired by digital camera or by Gel Doc™ EZ Imaging System with Image Lab™ Software 5.0 (Biorad). All images were edited and annotating by using Adobe Illustrator CS6.

### SELEX experiments

The template DNA library was prepared by Klenow reaction using a oligonucleotide harboring a 25-nt random sequence surrounded by 2 primer binding sites and a forward primer (carrying the T7 promoter region) (Supplementary Tables S1 and S2). This template DNA library was then purified (QIAquick Nucleotide Removal Kit, Qiagen), and 500 ng–1 µg used for the *in vitro* transcription reaction (T7 RiboMAX™ Express Large Scale RNA Production System, Promega). After removal of unincorporated nucleotides (mini Quick Spin RNA Columns, Roche), RNA was extracted with phenol/chloroform, ethanol-precipitated and finally resuspended in RNAse-free water (36 µL). Final RNA concentration was calculated by absorbance at 260 nm. Binding reactions were carried out in 200 µL of SELEX buffer (20 mM MOPS pH 7.0, 50 mM KCl, 5 mM MgCl_2_, 1 mM DTT, 0.1% Triton, 0.1 mM PMSF). Samples containing 40 pmol of GST-fused protein immobilized on GSH beads (GE Healthcare), 1–4 µg of heparin sulfate, and 0.4–3.2 nmol of RNA, were gently mixed at 4 °C for 60 min. Unbound RNA was removed by washing the beads (five times in 500 µL of SELEX buffer). RNA-protein complexes were dissociated by acidic elution in HCl-glycine 25 mM pH 2.2 (25 °C, 5 min). RNA was then purified (mini Quick Spin RNA Columns, Roche), ethanol-precipitated, and reverse-transcribed (SuperScript^®^ III Reverse Transcriptase, Invitrogen) using a complementary primer. Resulting cDNA was then amplified by 10 or 15 rounds of PCR and purified (Thermo Fisher Scientific, PCR clean up kit). A new RNA library was prepared using the amplified cDNA as a template for the following SELEX round. During the different cycles, selection stringency was intensified by increasing the RNA/protein ratios (round 1:10, round 2:20, round 3:30, round 4:40) together with the heparin amount (+1 µg/round). After 4 selection rounds, the final PCR products were subcloned into pJET vector (CloneJET PCR Cloning Kit, Thermo Fisher Scientific) and individual sequences were analyzed.

### Preparation of ssRNA oligonucleotides for the binding assays

ssRNA oligonucleotides (Supplementary Table S2) were purchased in dry form from Eurogentec (Belgium) and resuspended in DEPC water according to the instructions provided by the manufacturer. RNA samples were prepared by direct dilution of high concentrated stocks in experiment-specific buffers prior to use. All ssRNA oligonucleotides included the target sequence flanked by two (A)_3_ regions, to ensure target stability to degradation. BLI experiments on (ZF)_x2_ constructions, were carried out using 5′ biotinilated RNAs. For the MBP-(ZF)_x6_ chimeric protein, 3′biotinilated RNAs including a triethylenglycol (TEG) spacer were used (3′biotin-TEG), to reduce steric hindrance eventually induced by the MBP tag.

### Isothermal titration calorimetry (ITC)

Concentrated proteins and ssRNA oligonucleotides stocks were diluted into the ITC buffer (50 mM Tris pH 7.5, 150 mM NaCl, 1 mM DTT and 0.1 mM ZnCl_2_). Samples were kept RNase free by adding all of the time RNase inhibitor (0.4 u/µL) (Ribosafe Rnase Inhibitor, Bioline). All experiments were carried out by titrating proteins (100 μM) into RNA (10 μM), on a MicroCal i200 ITC microcalorimeter (GE Healthcare) at 25 °C. For each titration, an initial injection of 0.5 μL (discarded data) and 20 injections of 2 μL of titrant were made at 120 s intervals. Experimental data were corrected by subtraction of dilution heats from control experiments (protein titration into buffer) and finally analyzed (Origin7.0, MicroCal Software, Northampton, MA). The data were fitted to a 1:1 (one-site) model.

### Biolayer interferometry (BLI)

BLI experiments were performed at 30 °C in 96-well microplates (Pall), with agitation set to 1000 rpm, on a Octet HTX instrument (FortéBio, Pall). All assays were carried out in Tris 50 mM pH 7.5, NaCl 150 mM, DTT 1 mM, supplemented with BSA 0.1% and Tween 0.02% to minimize non-specific interactions. Proteins and RNAs were directly diluted in this buffer and RNase inhibitor (0.4 u/µL) (Ribosafe Rnase Inhibitor, Bioline) was added to ensure RNase free conditions. For all experiments on (ZF)_x2_ constructions, biotinylated RNAs (0.5 µg/mL) were immobilized on streptavidin-coated biosensors (Pall) for 300 s. The assays conducted on the (ZF)_x6_ chimeric protein, were performed with a loading RNA concentration of 0.125 µg/mL and a duration of 500 s in order to increase the spacing between immobilized RNAs and prevent possible dimerization. Biosensor tips were then saturated in biocytin (10 µg/mL) for 500 s to block any remaining free streptavidin, and equilibrated in the experimental buffer for 180 s prior to binding assessment. We tested different protein concentrations ranging from 1.5 to 0.31 µM (1.3-fold dilution series) and from 5 to 0.44 µM (1.5-fold dilution series) for the (ZF)_x2_ and (ZFs)_x6_ chimeric proteins, respectively. In all cases, binding was performed for 10 s and dissociation in the experimental buffer for 20 s. Recorded data were corrected by subtraction from the measurements of a reference sensor immobilized with biotinylated RNA (baseline drift). Data were analyzed using Octet software, version 8.0 (Pall) and fitted using a 1:1 interaction model. A single set of kinetic parameters was obtained each time for all tested concentrations by nonlinear least-squares fitting.

The Microlab STAR liquid handling workstation (Hamilton) and the Octet HTX instrument (FortéBio, Pall) are available at the Robotein^®^ high-throughput platform for protein production and analysis (www.robotein.ulg.ac.be).

### Sequence conservation analysis of putative RNA-binding RanBP2-type ZFs

In order to select the putative RNA-binding RanBP2-type ZFs, we extracted from the RanBP2-type family (accession code pfam00641) all protein sequences showing an aromatic residue at position 17. This initial database was reduced to 1704 entries by eliminating redundancy. An additional correction was carried out by removing sequences not displaying to the Trp-X-Cys-X2-4-Cys-X3-Asn-X6-Cys- consensus. The resulting 1526 sequences were analyzed by WebLogo software^51^, providing a graphical representation of the developed sequence alignment.

### RNA-binding bacterial assay

In these experiments, we employed the pACYDuet-1 vector for the co-expression of two genes, each under the control of IPTG inducible T7 promoter. We designed a sequence coding for the chimeric (ZF)_x6_ array and a C-terminal MBP-tag, fused by a 5-amino acids GS-linker. Both PCR-amplified (ZF)_x6_ and MBP DNA sequences harbored at their ends the BamHI (5′) and EcoRI (3′) restriction sites. The full sequence coding for (ZF)_x6_MBP was assembled between the restriction sites NcoI and HindIII (first MCS) of the pACYDuet-1 vector. Furthermore, we redesigned the second expression cassette of the vector to accommodate the target sequence of the (ZF)_x6_ array upstream the RBS (+33 to +53). Downstream the RBS and between the EcoRV and XhoI restriction sites, we assembled the sequence coding for the green fluorescent protein unstable variant GFP LVA^35^. Both target and GFP LVA sequences were commercially purchased as gBlocks^®^ Gene Fragments (Integrated DNA Technologies, IDT). The pACYDuet-(ZF)_x6_MBP-target-GFP vector was obtained by assembling multiple DNA fragments in a single step using the NEBuilder^®^ High-Fidelity Master Mix (New England Biolabs), following the manufacturer’s instructions. In addition, we assembled a control plasmid pACYDuet-(ZF)_x6_MBP-GFP that carried no target sequence. From these vectors, we excised either the (ZF)_x6_ or the MBP coding sequence by single digestion. Then, the digested plasmids were gel-extracted, purified and self-ligated. Using this approach, we obtained both negative control (pACYDuet-MBP-target-GFP) and positive control (pACYDuet-(ZF)_x6_-target-GFP) from digestion of the pACYDuet-(ZF)_x6_MBP-target-GFP (Supplementary Fig. S10). Additional controls were generated by digestion of the pACYDuet-(ZF)_x6_MBP-GFP (pACYDuet-MBP-GFP and pACYDuet-(ZF)_x6_-GFP), therefore providing a full set of control vectors with no target sequence (Supplementary Fig. S10). The integrity of each plasmid was verified by DNA sequencing. Genetic constructions were introduced in *E. coli* Single Step KRX cells (Promega) by heat-shock transformation. This strain ensured a dramatic control of the GFP LVA expression, thanks to the T7 RNA polymerase driven by a rhamnose promoter. For each transformed construct, various dilutions of a single colony were cultured in 160 µL of LB with chloramphenicol (30 μg/mL) in a 96 well plate at 37 °C with shacking. When an OD of 0.3–0.6 was achieved, bacterial dilutions exhibiting similar density were selected for culture inoculation. We prepared the assay plate (Greiner Bio One, Belgium) by diluting 2 µL of each cell suspension in 160 µL of LB supplemented with chloramphenicol (30 μg/mL). Cells were cultured at 37 °C with shacking in a plate reader TECAN infinite^®^ 200 PRO (Tecan Group Ltd., Switzerland). When cultures reached an OD of 0.3–0.35, protein expression was induced by adding 0.1% L-rhamnose and 0.5 mM IPTG, at 37 °C. As for all ZF productions, 0.1 mM ZnCl_2_ was added as well. The OD measurements were performed at 600 nm with a bandwidth of 9 nm. Fluorescence measurements were carried out using a excitation wavelength of 485 nm with a bandwidth of 9 nm, and the emission wavelength of 525 nm with a bandwidth of 20 nm. For each measurement,10 reads were performed every 15 min, with gain set to 50; lag, integration and settle time were set to 0 μs, 20 μs and 0 ms, respectively.

## Supplementary information

tables and figures
