# Supplementary material for: Exploring the suitability of RanBP2-type Zinc Fingers for RNA-binding protein design
Source: Sci Rep. 2019 Feb 21;9:2484. doi: 10.1038/s41598-019-38655-y (PMC6384913; doi:10.1038/s41598-019-38655-y)

Exploring the suitability of RanBP2-type Zinc Fingers for RNA-binding protein design.

Simona De Franco[1], Julie Vandenameele[1], Alain Brans[1], Olivier Verlaine[1], Katerina Bendak[2], Christian Damblon[3], André Matagne[1], David J. Segal[4], Moreno Galleni[1], Joel P. Mackay[5], and Marylène Vandevenne[1]*

Supplementary Information - Tables

ssDNA oligonucleotide	Sequence
Template library	TCCCGCTCGTCGTCTNNNNNNNNNNNNNNNNNNNNNNNNNNCCGCATC GTCCTCCCT
Forward primer	GAAATTAATACGACTCACTATAGGGAGGACGATGCGG
Reverse primer	TCCCGCTCGTCGTCTG

Table S1. Sequences of the DNA oligonucleotides used in SELEX experiments. The T7 promoter region included in the forward primer is shown in red.

ssRNA sequence	Modification
AAAGGUGGUAAA	none
AAAGGAGGAAAA	none
AAAAAAAAAAAA	none
AAAGGUGGUAAA	5’ Biotin
AAAGGAGGAAAA	5’ Biotin
AAAAAAAAAAAA	5’ Biotin
AAAAGUAGUAAA	5’ Biotin
AAAGAUGAUAAA	5’ Biotin
AAAAGAAGAAAA	5’ Biotin
AAAGUAGUAAAA	5’ Biotin
AAAGGUUAUUGGUGCCGGGUAUUAAA	3’ Biotin-TEG
AAAAAAAAAAAAAAAAAAAAAAAAAA	3’ Biotin-TEG
AAANNNNNNNNNNNNNNNNNNNNAAA	3’ Biotin-TEG

Table S2. Sequences of the ssRNA oligonucleotides used in the ITC and BLI experiments.

GST-$(ZF)_2$	[GST-ZF_2] (µM)	[Zn] (µM)	[Zn]/[GST-ZF_2]	[Zn]/ [ZF_2]	f_{dizinc}*
GST-$(FUS-ZF)_2$	15,4	35,4 ± 0,1	2,3	1,5	0,75
GST-$(EWS-ZF)_2$	15,4	34,8 ± 2,9	2,3	1,5	0,75
GST-$(ZRANB1-B-ZF)_2$	15,6	29,5 ± 2,1	1,9	1,1	0,54
GST-(ZRANB2)-(ZF1-ZF2)	15,4	26,0 ± 6	1,7	0,9	0,45
GST-$(ABI3-5-ZF)_2$	15,3	32,1 ± 0,3	2,1	1,3	0,65
GST-$(T0B2.5-ZF)_2$	15,6	53,8 ± 2,2	3,45	2,65	1,32
GST-$(hRBM10)_2$	15,4	30,8 ± 1,3	2	1,200	0,60
GST-$(mRBM10)_2$	15,6	33,2 ± 0,2	2,13	1,33	0,66
GST	15,4	12,3± 1,9	0,8	/	/

Table S3. Inductively coupled plasma mass spectrometry (ICP-MS) experiments performed on the GST-$(ZF)_2$ fusion proteins. The proteins were all dialyzed in 20 mM ammonium acetate pH 7 and then mineralized with 10% nitric acid. All the protein concentrations were set to 15 µM. The zinc concentration was measured at 472.2 nm based on a calibration curve performed with a stock solution of known zinc concentration. The GST alone was used as a negative control. The dialysis buffer (used as a blank) exhibited slightly negative values for the zinc concentration. The stoichiometry (zinc)/(GST-ZF_2) represents the ratio between the concentration of zinc determined by ICP-MS and the GST fusion protein concentration. The stoichiometry (zinc)/(Zf_2) was obtained by subtracting the stoichiometry observed for the GST alone to the stoichiometry values reported for the GST-ZF_2 fusion proteins. *The last column represents the fraction of proteins (ZF_2) that coordinates 2 zincs (fdizinc).

Supplementary Information -Figures S1- S12

Figure S1. **Schematic representation of the different constructs employed in this work**. Amino acid sequences of the ZF domains and the linker region are shown in blue and red, respectively. A) RanBP2-type $(ZF)_{x2}$ variants used in the SELEX experiment. B) RBM10 $(ZF)_{x2}$ natural and mutated variants (mutated residues are underlined). C) $(ZF)_{x6}$ chimeric protein.

Figure S2. ZRANB2-(ZF1,2) purification by size exclusion chromatography. Panel A shows the chromatogram, namely the variation of UV absorbance (blue) over volume (black) and collected fractions (red). The first peak resulted from GST-ZRANB2-(ZF1,2) and GST co-elution, and the second peak from ZRANB2-(ZF1,2) elution. The third peak corresponded to small-size impurities, as confirmed by SDS-PAGE analysis. Panel B shows the SDS-PAGE analysis of loading sample and fractions from collected peaks (same numbering as the chromatogram); the full-length gel is presented in Supplementary Figure S10. Electrophoresis was performed on 4-20% precast polyacrylamide gels, using unstained protein ladder (Pierce, Thermo Scientific, USA).

Figure S3. **ITC data for $(ZF)_{x2}$ protein: RNA interactions.** Raw data and integrated enthalpy changes are plotted as a function of RNA: $(ZF)_{x2}$ molar ratios in top and bottom panels, respectively. The data fits (to a 1:1 binding model) are also shown.

Figure S4. BLI data for $(ZF)_{x2}$ protein: RNA interactions. Reported graphs refer to single experiments. Sensograms are shown in blue or green whereas the corresponding fits (1:1 model) are red.

Figure S5. Kinetic characterization of hRBM10, mRBM10, N4D and M26V mutants ZFs. BLI data collected for all the studied RBM10 variants/mutants showing their interaction with a $(GGG)_{x2}$ sequence. Reported graphs refer to single experiments. Sensograms and corresponding fits (1:1 model) are blue and red, respectively.

Figure S6. Discrimination ability of $(ZF)_{x2}$ proteins. BLI data collected for any modification of the third base in target RNAs. **Reported graphs refer to single experiments. Sensograms and corresponding fits (1:1 model) are blue and red, respectively**

Figure S7. A) SDS-PAGE analysis of the MBP-$(ZF)_{x6}$ purification by amylose affinity chromatography. B and C. Purification step performed by Size Exclusion Chromatrography (SEC). B represents the elution chromatogram showing the A^{280} (blue), the elution volume (black) and the collected fractions (red). C shows the SDS-PAGE analysis of the loaded sample and collected fractions. Full-length gels are presented in Supplementary Figure S11, S12.

Figure S8. Plasmid preparation for the RNA-binding *in vivo* assay. All plasmids employed in our assay derived from the firstly assembled pACYDuet-$(ZF)_{x6}$MBP-target-GFP and pACYDuet-$(ZF)_{x6}$MBP-GFP. From these vectors, either the $(ZF)_{x6}$ or the MBP coding sequence was excised by single digestion. Then, digested plasmids were purified and self-ligated. Using this approach, we obtained the pACYDuet-MBP-target-GFP, pACYDuet-$(ZF)_{x6}$-target-GFP, pACYDuet-MBP-GFP and pACYDuet-$(ZF)_{x6}$-GFP) vectors.

Figure S9. Architectures of full-length proteins containing RanBP2-type ZF(s). Only proteins whose RanBP2 type zinc finger(s) is (are) able to interact with RNA are presented. In the ABI3-5 Sup and RBM10 full-length proteins, the RanBP2-type ZFs are surrounded by RNA recognition motifs (RRMs). Notably, these proteins are characterized by a larger number of RNA binding domains compared to ZRANB2, EWS and Fus proteins. This observation could explain the evolution of different specificities between the ZRANB2, EWS, FUS versus ABI3-5 Sup, RBM10 RanBP2-type ZFs.

Figure S10. Full-length gel displayed in Supplementary Figure S2B. Supplementary Figure S2B shows from lane 2, which corresponds to the sample of digested GST-ZRANB2-(ZF1,2) loaded on a SEC column. Lane 1 corresponds to the same sample before filtration on a 0.22 nm membrane and was omitted from Supplementary Figure S2B for simplicity.

Figure S11. Full-length gel displayed in Supplementary Figure S7A. Gel lanes are detailed in Figure S7A.

Figure S12. Full-length gel displayed in Supplementary Figure S7C. Gel lanes are detailed in Figure S7C.

Figure S1

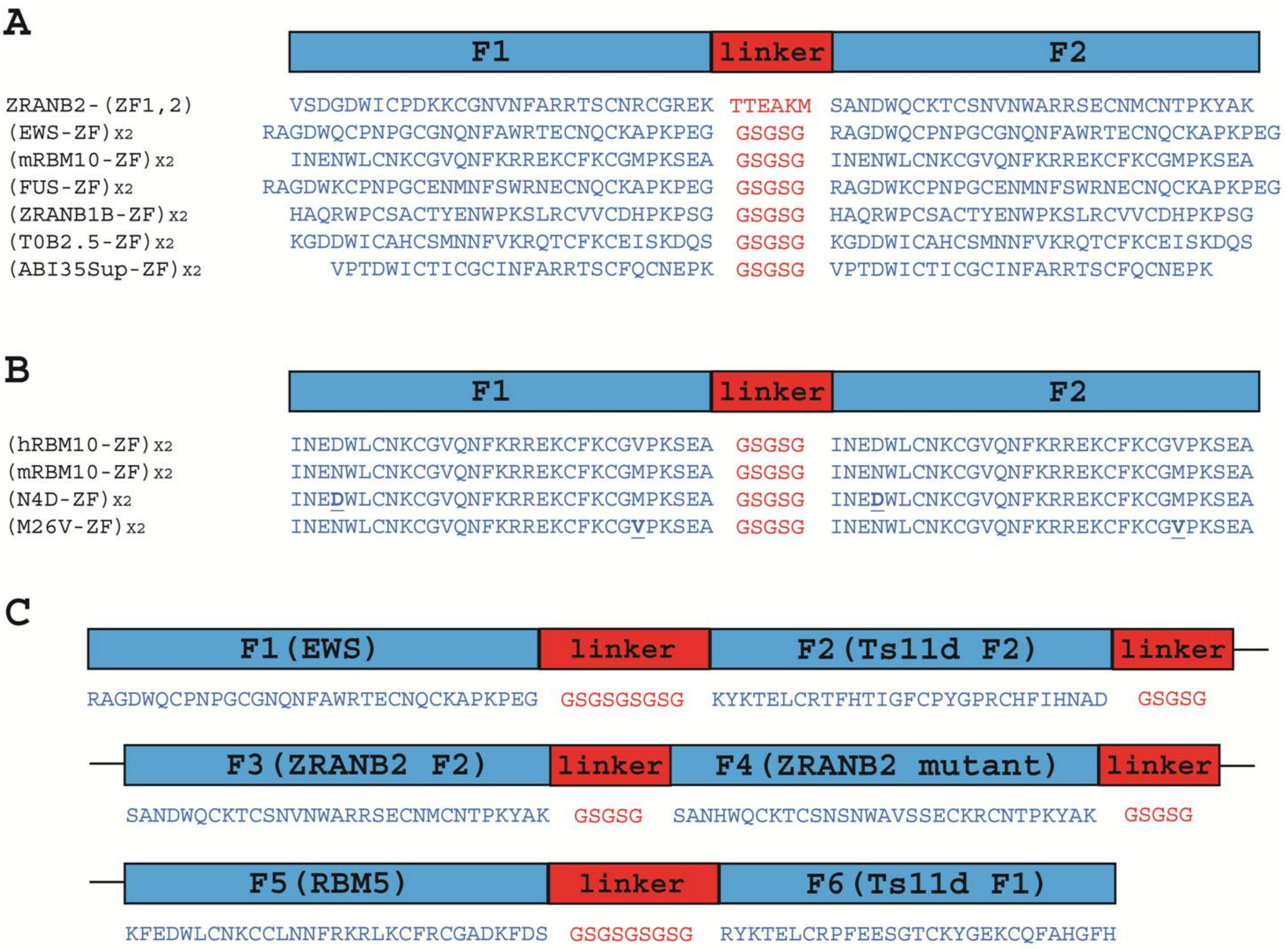
A
F1
linker
F2
ZRANB2-(ZF1,2) VSDGDWICPDKKCGNVNFARRTSCNRCGREK TTEAKM SANDWQCKTCSNVNWARRSECNMCNTPKYAK
(EWS-ZF)x2 RAGDWQCPNPGCGNQNFAWRTECNQCKAPKPEG GSGSG RAGDWQCPNPGCGNQNFAWRTECNQCKAPKPEG
(mRBM10-ZF)x2 INENWLCNKCGVQNFKRREKCFKCGMPKSEA GSGSG INENWLCNKCGVQNFKRREKCFKCGMPKSEA
(FUS-ZF)x2 RAGDWKCPNPGCENMNFSWRNECNQCKAPKPEG GSGSG RAGDWKCPNPGCENMNFSWRNECNQCKAPKPEG
(ZRANB1B-ZF)x2 HAQRWPCSACTYENWPKSLRCVVCDHPKPSG GSGSG HAQRWPCSACTYENWPKSLRCVVCDHPKPSG
(T0B2.5-ZF)x2 KGDDWICAHCSMNNFVKRQTCFKCEISKDQS GSGSG KGDDWICAHCSMNNFVKRQTCFKCEISKDQS
(ABI35Sup-ZF)x2 VPTDWICTICGCINFARRTSCFQCNEPK GSGSG VPTDWICTICGCINFARRTSCFQCNEPK
B
F1
linker
F2
(hRBM10-ZF)x2 INEDWLCNKCGVQNFKRREKCFKCGVPKSEA GSGSG INEDWLCNKCGVQNFKRREKCFKCGVPKSEA
(mRBM10-ZF)x2 INENWLCNKCGVQNFKRREKCFKCGMPKSEA GSGSG INENWLCNKCGVQNFKRREKCFKCGMPKSEA
(N4D-ZF)x2 INEDWLCNKCGVQNFKRREKCFKCGMPKSEA GSGSG INEDWLCNKCGVQNFKRREKCFKCGMPKSEA
(M26V-ZF)x2 INENWLCNKCGVQNFKRREKCFKCGVPKSEA GSGSG INENWLCNKCGVQNFKRREKCFKCGVPKSEA
C
F1(EWS)
linker
F2(Ts11d F2)
linker
RAGDWQCPNPGCGNQNFAWRTECNQCKAPKPEG GSGSGSGSG KYKTELCRTFHTIGFCPYGPRCHFIHNAD GSGSG
F3(ZRANB2 F2)
linker
F4(ZRANB2 mutant)
linker
SANDWQCKTCSNVNWARRSECNMCNTPKYAK GSGSG SANHWQCKTCSNSNWAVSSECKRCNTPKYAK GSGSG
F5(RBM5)
linker
F6(Ts11d F1)
KFEDWLCNKCCLNNFRKRLKCFRCGADKFDS GSGSGSGSG RYKTELCRPFEESGTCKYGEKCQFAHGFH

Figure S2

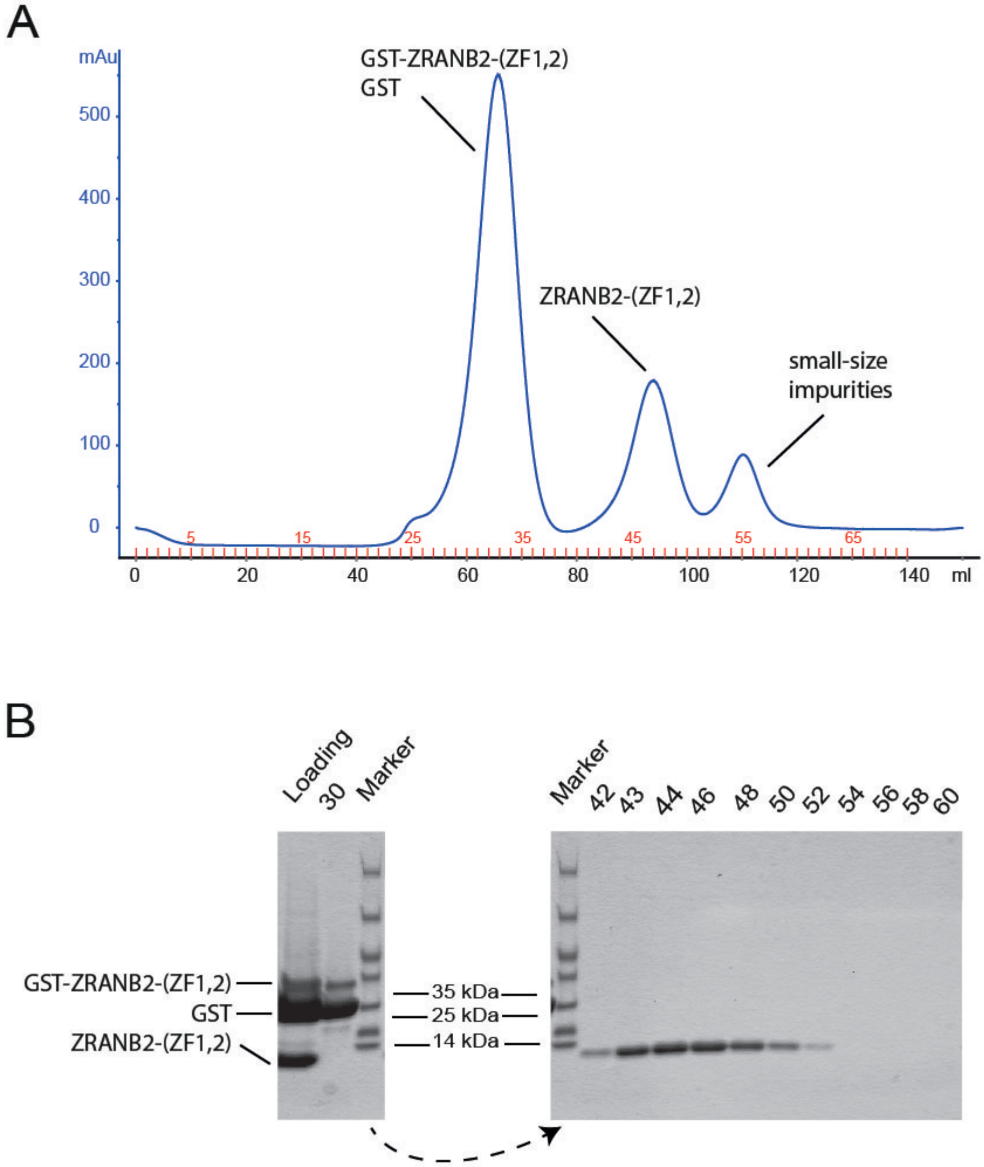
A
mAu
500
400
300
200
100
0
GST-ZRANB2-(ZF1,2)
GST
ZRANB2-(ZF1,2)
small-size
impurities
5
15
25
35
45
55
65
0
20
40
60
80
100
120
140
ml
B
Loading
30
Marker
Marker
42
43
44
46
48
50
52
54
56
58
60
GST-ZRANB2-(ZF1,2)
GST
ZRANB2-(ZF1,2)
35 kDa
25 kDa
14 kDa

Figure S3

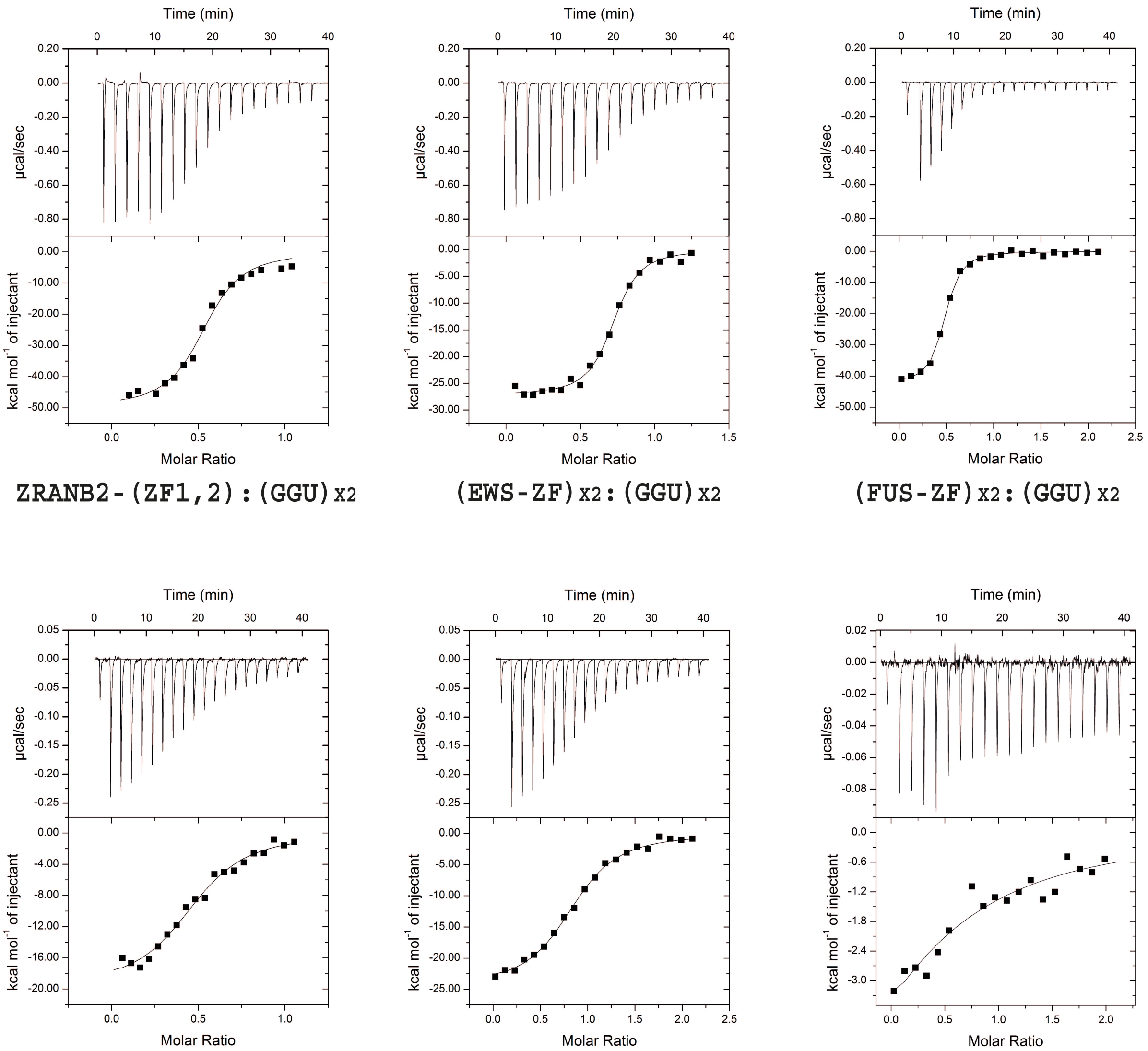

ZRANB2-(ZF1,2):(GGU)x2 | (EWS-ZF)x2:(GGU)x2 | (FUS-ZF)x2:(GGU)x2

(ABI3-5Sup-ZF)x2:(GGA)x2 | (ABI3-5Sup-ZF)x2:(GGU)x2 | (mRBM10-ZF)x2:(GGU)x2

Figure S4

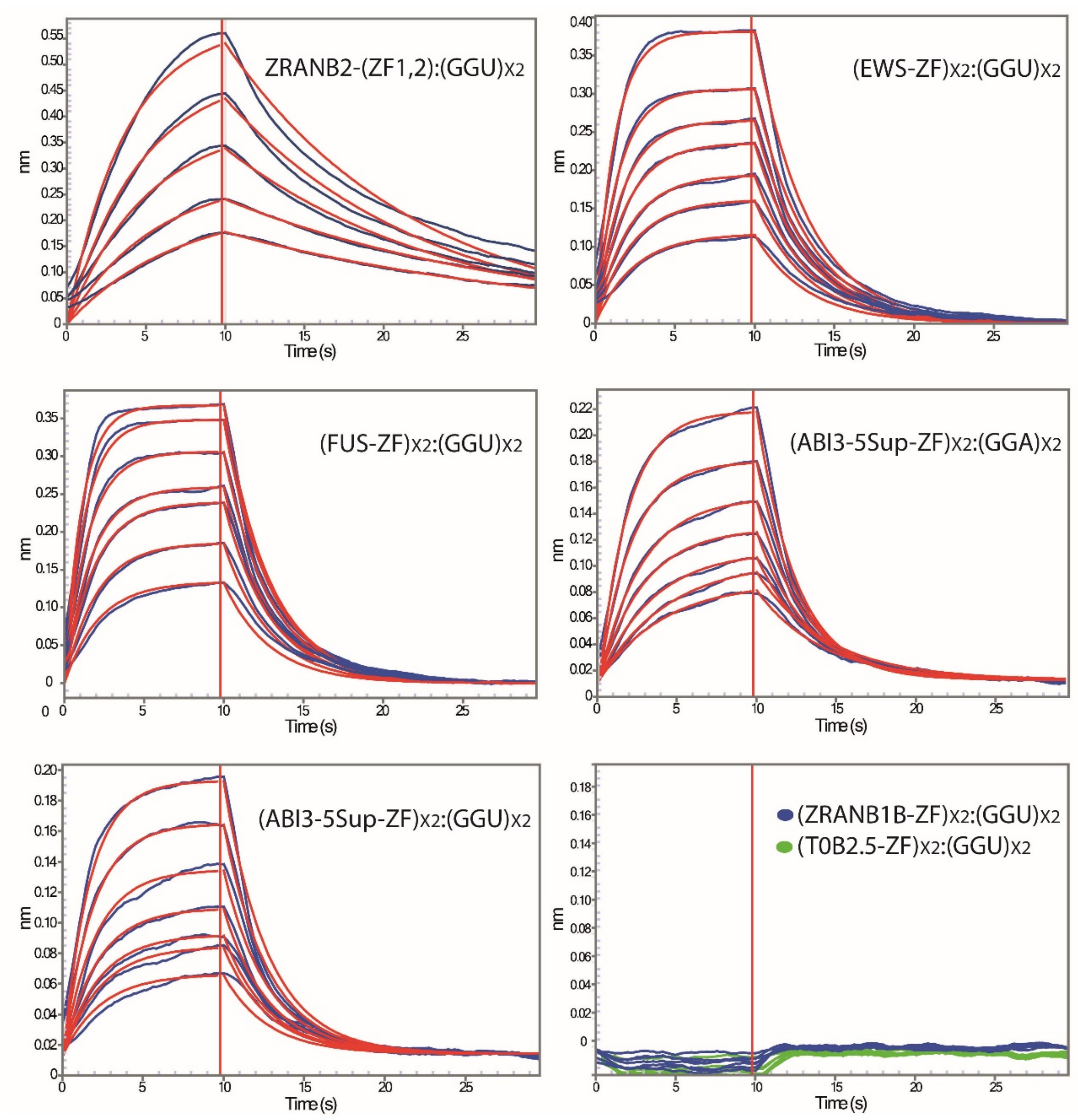

ZRANB2-(ZF1,2):(GGU)x2
(EWS-ZF)x2:(GGU)x2
(FUS-ZF)x2:(GGU)x2
(ABI3-5Sup-ZF)x2:(GGA)x2
(ABI3-5Sup-ZF)x2:(GGU)x2
(ZRANB1B-ZF)x2:(GGU)x2
(T0B2.5-ZF)x2:(GGU)x2
nm
Time (s)

Figure S5

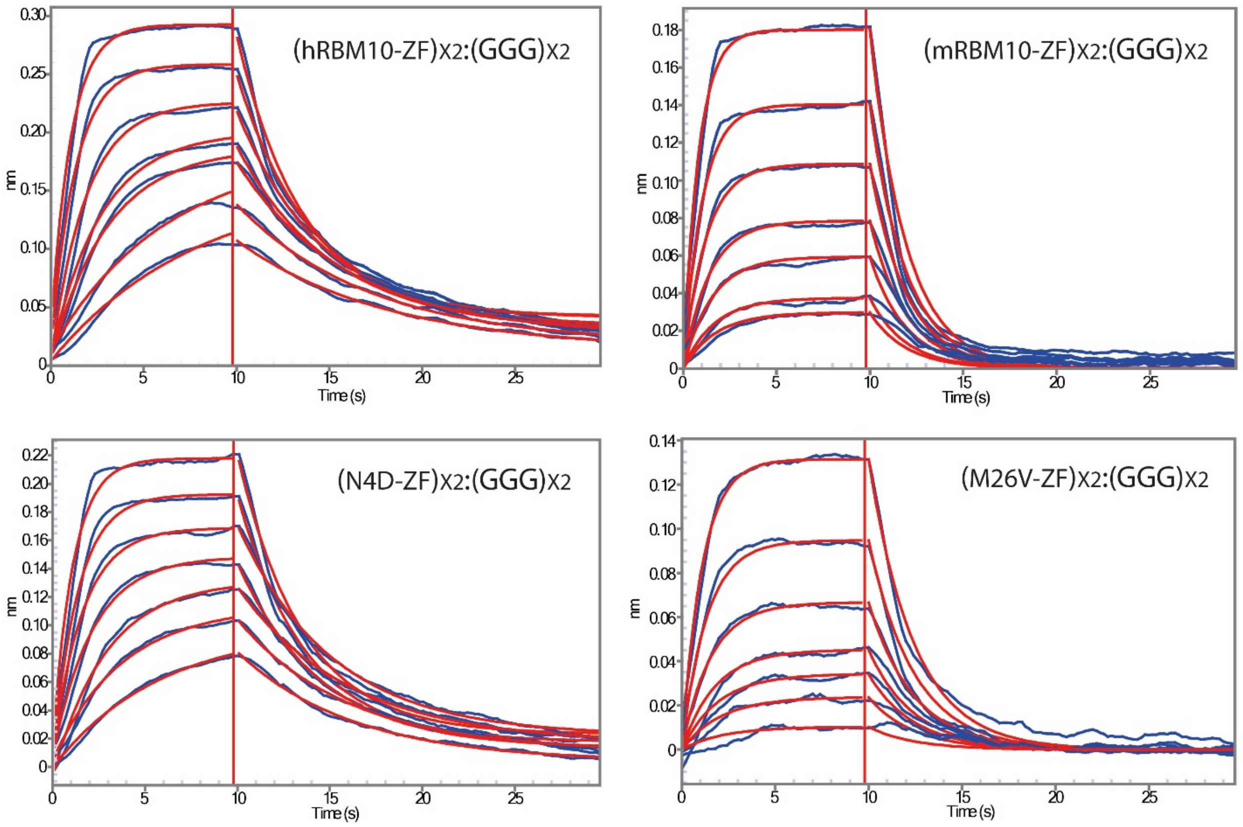
(hRBM10-ZF)X2:(GGG)X2
(mRBM10-ZF)X2:(GGG)X2
(N4D-ZF)X2:(GGG)X2
(M26V-ZF)X2:(GGG)X2
nm
Time (s)
0.30
0.25
0.20
0.15
0.10
0.05
0
0.18
0.16
0.14
0.12
0.10
0.08
0.06
0.04
0.02
0.22
0
5
10
15
20
25

Figure S6

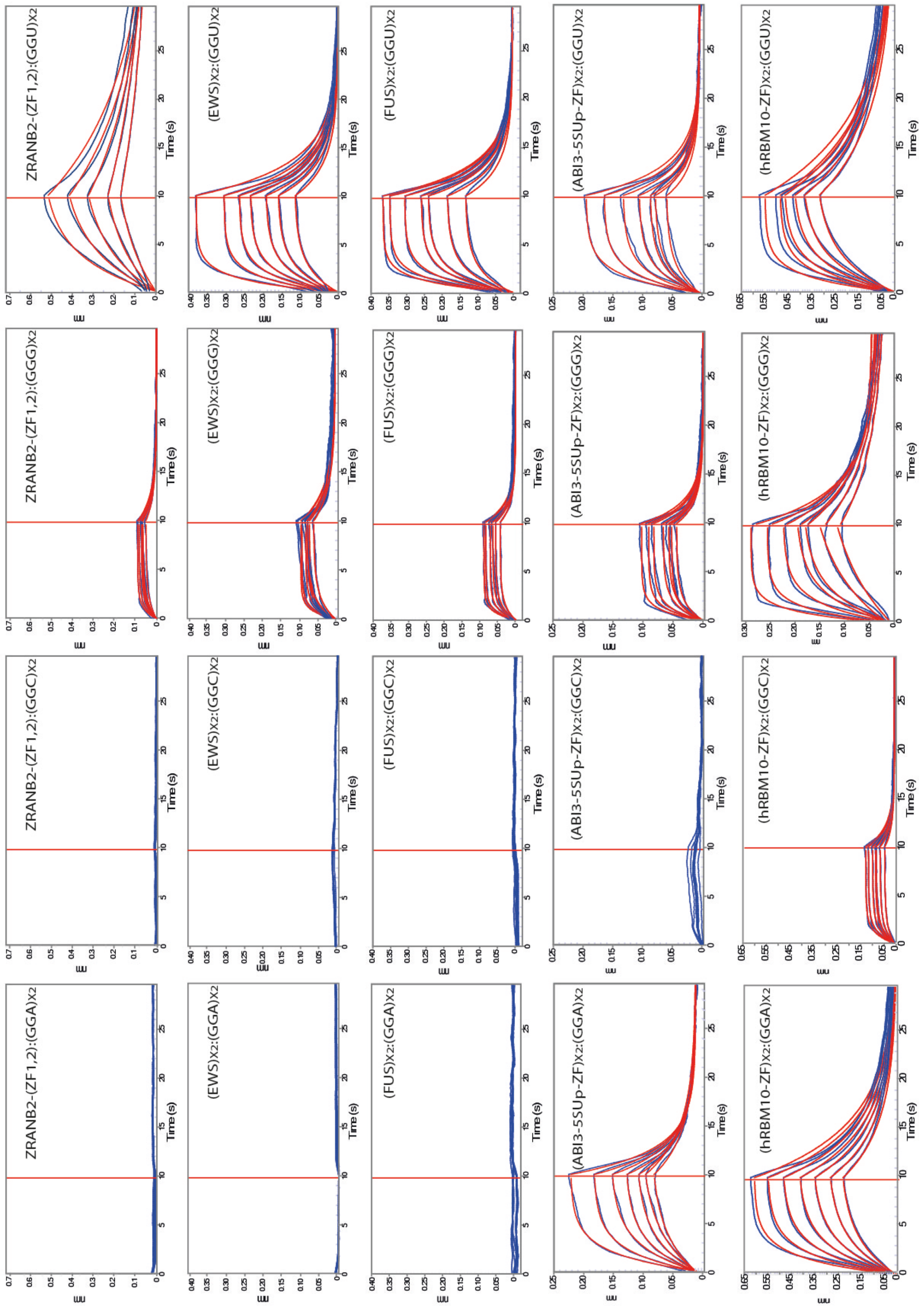
ZRANB2-(ZF1,2):(GGU)x2
ZRANB2-(ZF1,2):(GGG)x2
ZRANB2-(ZF1,2):(GGC)x2
ZRANB2-(ZF1,2):(GGA)x2
(EWS)x2:(GGU)x2
(EWS)x2:(GGG)x2
(EWS)x2:(GGC)x2
(EWS)x2:(GGA)x2
(FUS)x2:(GGU)x2
(FUS)x2:(GGG)x2
(FUS)x2:(GGC)x2
(FUS)x2:(GGA)x2
(ABI3-5SUp-ZF)x2:(GGU)x2
(ABI3-5SUp-ZF)x2:(GGG)x2
(ABI3-5SUp-ZF)x2:(GGC)x2
(ABI3-5SUp-ZF)x2:(GGA)x2
(hRBM10-ZF)x2:(GGU)x2
(hRBM10-ZF)x2:(GGG)x2
(hRBM10-ZF)x2:(GGC)x2
(hRBM10-ZF)x2:(GGA)x2
Time (s)

Figure S7

A

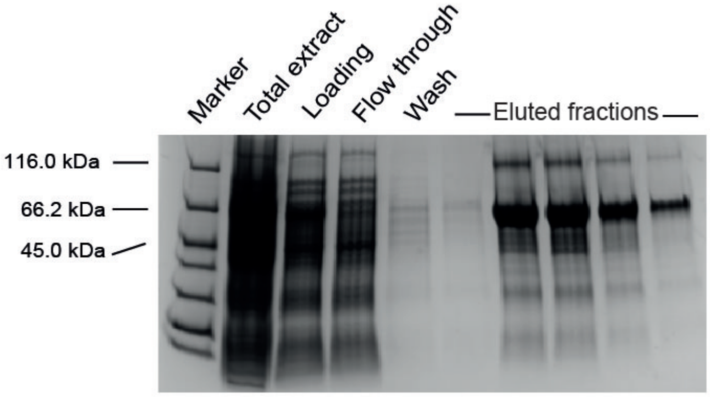

B

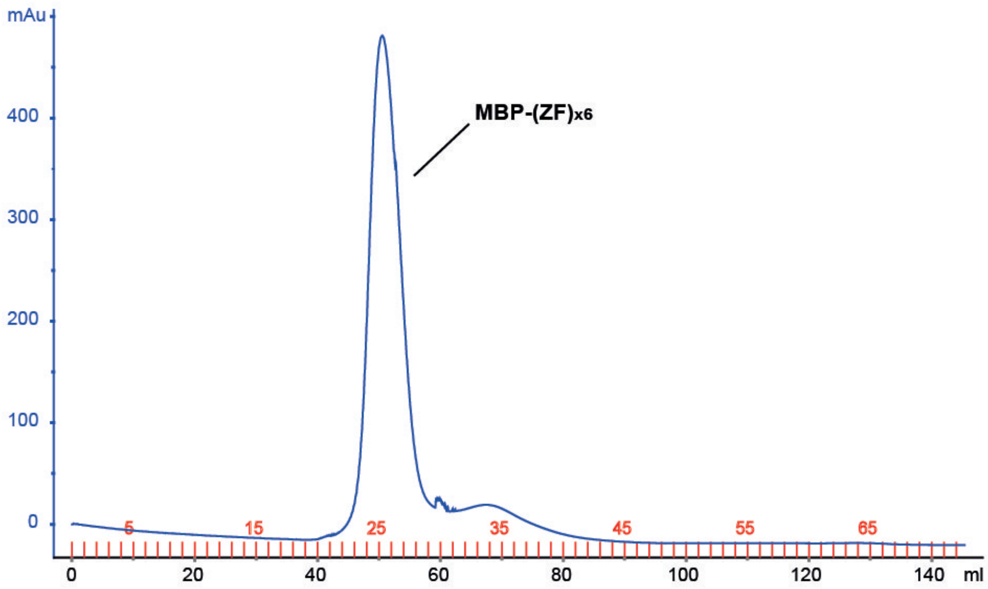

C

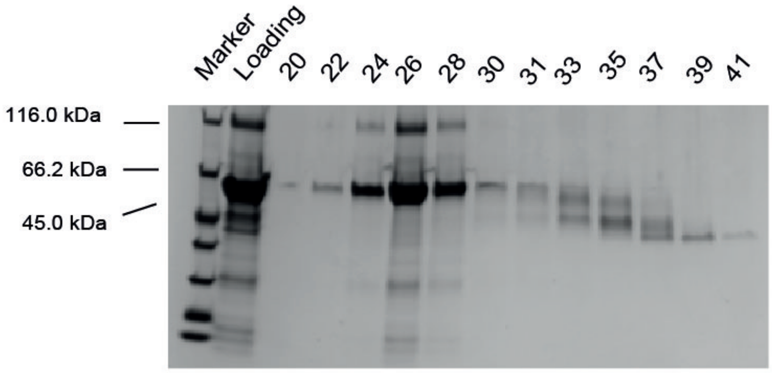

Figure S8

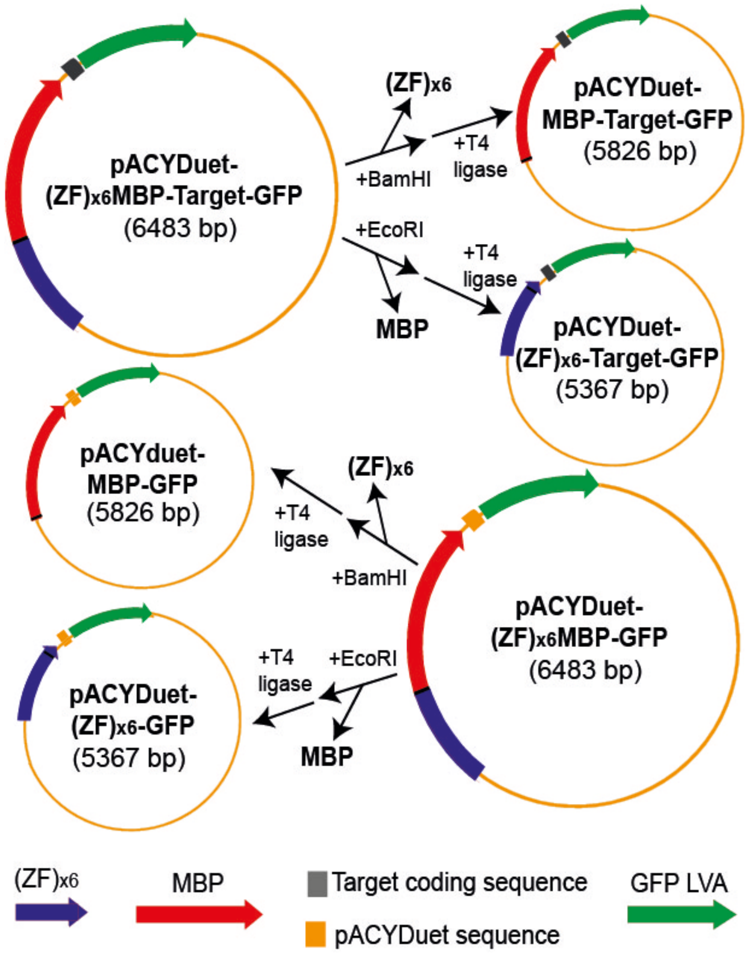

pACYDuet-
(ZF)x6MBP-Target-GFP
(6483 bp)
(ZF)x6
+BamHI
+T4
ligase
pACYDuet-
MBP-Target-GFP
(5826 bp)
+EcoRI
+T4
ligase
MBP
pACYDuet-
(ZF)x6-Target-GFP
(5367 bp)
pACYduet-
MBP-GFP
(5826 bp)
(ZF)x6
+T4
ligase
+BamHI
pACYDuet-
(ZF)x6MBP-GFP
(6483 bp)
+T4
ligase
+EcoRI
MBP
pACYDuet-
(ZF)x6-GFP
(5367 bp)
(ZF)x6
MBP
Target coding sequence
pACYDuet sequence
GFP LVA

Figure S9

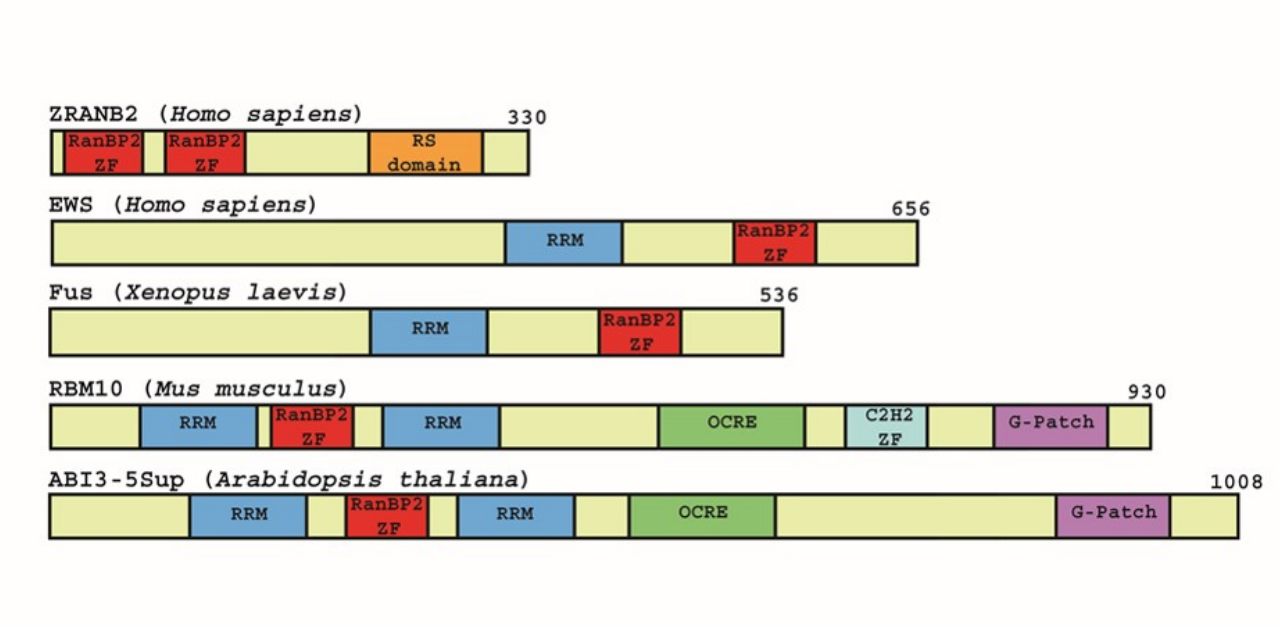

ZRANB2 (*Homo sapiens*)
330
RanBP2 ZF
RanBP2 ZF
RS domain
EWS (*Homo sapiens*)
656
RRM
RanBP2 ZF
Fus (*Xenopus laevis*)
536
RRM
RanBP2 ZF
RBM10 (*Mus musculus*)
930
RRM
RanBP2 ZF
RRM
OCRE
C2H2 ZF
G-Patch
ABI3-5Sup (*Arabidopsis thaliana*)
1008
RRM
RanBP2 ZF
RRM
OCRE
G-Patch

.

Figure S10

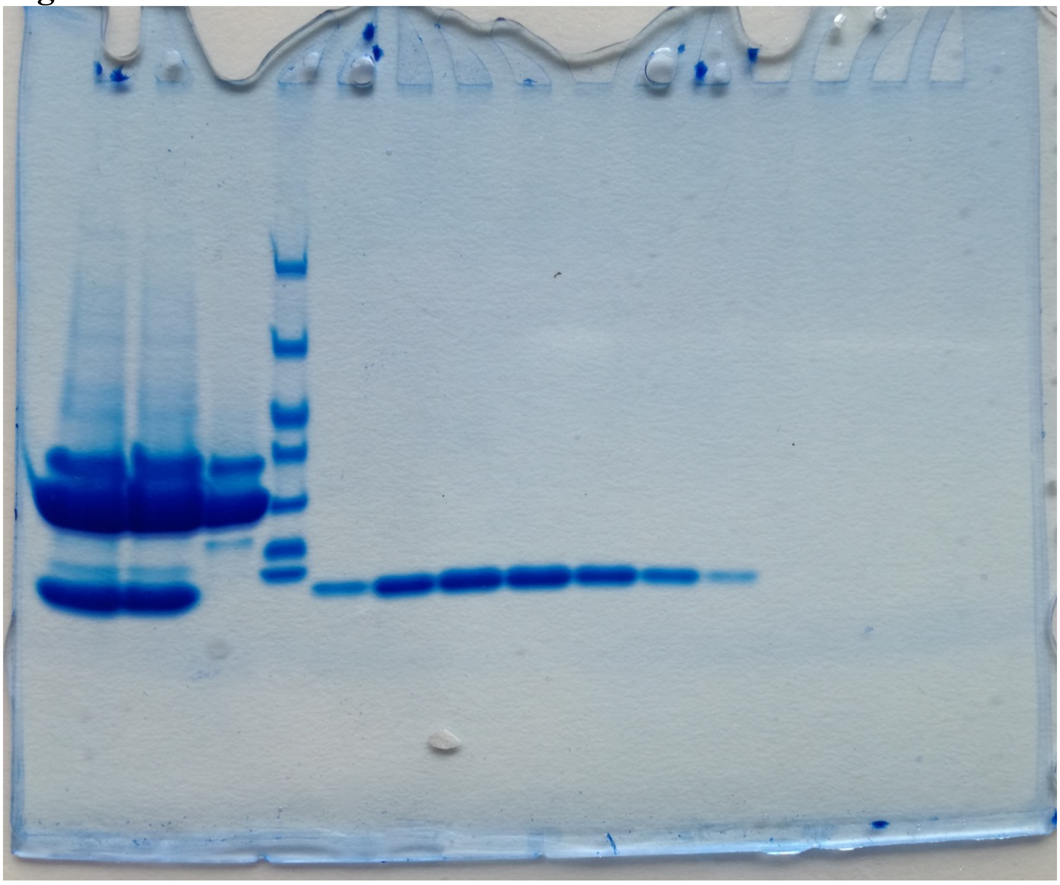

Figure S11

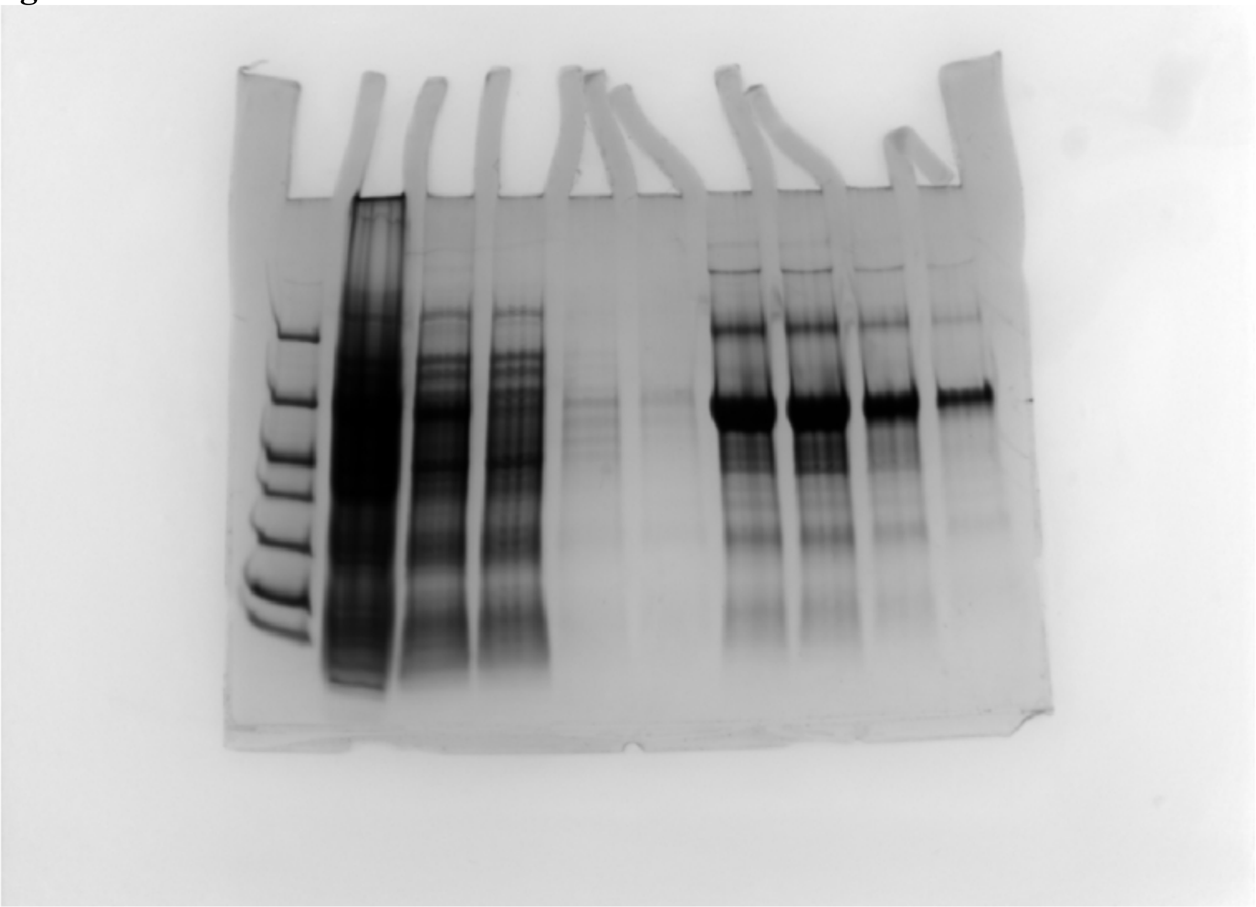

Figure S12

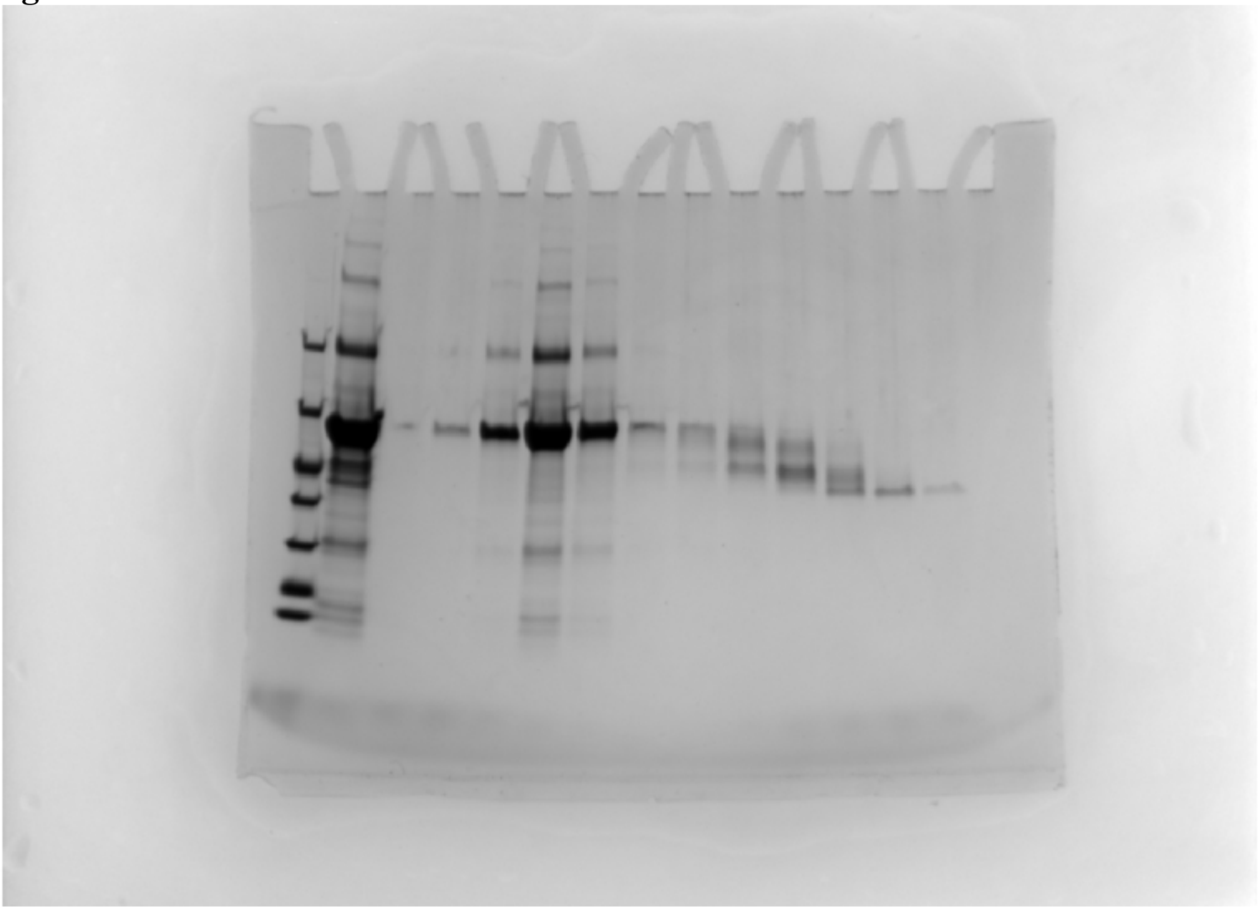

Supplement: Supplementary file 1 — tables and figures [file 41598_2019_38655_MOESM1_ESM.pdf]
